# Cooperative microbial metabolism enhances tryptophan-mediated insecticide detoxification in the fall armyworm

**DOI:** 10.1093/ismejo/wraf237

**Published:** 2025-10-24

**Authors:** Yunhua Zhang, Wujia Mo, Keyi Chen, Yichen Ding, Kaikai Mao, Hu Wan, Jizhong Zhou, Feng Ju

**Affiliations:** Zhejiang Provincial Key Laboratory of Intelligent Low-Carbon Biosynthesis, Research Center for Industries of the Future, School of Engineering, Westlake University, Hangzhou, Zhejiang 310030, China; Institute of Advanced Technology, Westlake Institute for Advanced Study, 18 Shilongshan Road, Hangzhou, Zhejiang 310024, China; Current Affiliation: College of Life and Environmental Sciences, Hangzhou Normal University, Hangzhou, 311121, China; Zhejiang Provincial Key Laboratory of Intelligent Low-Carbon Biosynthesis, Research Center for Industries of the Future, School of Engineering, Westlake University, Hangzhou, Zhejiang 310030, China; Zhejiang Provincial Key Laboratory of Intelligent Low-Carbon Biosynthesis, Research Center for Industries of the Future, School of Engineering, Westlake University, Hangzhou, Zhejiang 310030, China; Zhejiang Provincial Key Laboratory of Intelligent Low-Carbon Biosynthesis, Research Center for Industries of the Future, School of Engineering, Westlake University, Hangzhou, Zhejiang 310030, China; Guangxi Key Laboratory of Agro-Environment and Agric-Products Safety, College of Agriculture, Guangxi University, Nanning, Guangxi 530004, China; State Key Laboratory of Agricultural Microbiology, Huazhong Agricultural University, Wuhan, Hubei 430070, China; Institute for Environmental Genomics, University of Oklahoma, Norman, OK 73019, United States; Zhejiang Provincial Key Laboratory of Intelligent Low-Carbon Biosynthesis, Research Center for Industries of the Future, School of Engineering, Westlake University, Hangzhou, Zhejiang 310030, China; Institute of Advanced Technology, Westlake Institute for Advanced Study, 18 Shilongshan Road, Hangzhou, Zhejiang 310024, China; Westlake Laboratory of Life Sciences and Biomedicine, Center for Infectious Disease Research, School of Life Sciences, Westlake University, Hangzhou, Zhejiang 310024, China; Center for Future Foods, Mu Yuan Laboratory, Zhengzhou, Henan 450016, China

**Keywords:** insecticide resistance, microbiome and gut symbionts, tryptophan metabolism, indoleacetic acid (IAA), fall armyworm (*Spodoptera frugiperda*)

## Abstract

The fall armyworm, *Spodoptera frugiperda*, is a major global agricultural pest, known for its rapid evolution of insecticide resistance. Although host genetic adaptation contributes to this trait, the role of gut symbiont-mediated metabolic pathways in promoting resistance remains poorly understood. Here, we show that besides direct biodegradation, a generalist symbiont *Enterococcus casseliflavus* EMBL-3 indirectly promotes chlorantraniliprole resistance by compensating for tryptophan deficiency in a maize-based diet. Metabolomics and isotope tracing identify EMBL-3 as the primary producer of tryptophan, which is subsequently converted by co-resident microbes to indoleacetic acid. Indoleacetic acid activates the aryl hydrocarbon receptor, leading to upregulation of UDP-glucuronosyltransferase, a detoxification enzyme essential for chlorantraniliprole resistance, as confirmed by CRISPR/Cas9 knockout. This tripartite EMBL-3–indoleacetic acid–UDP-glucuronosyltransferase axis defines a hierarchical symbiont-host metabolic network driving chlorantraniliprole resistance. Our findings provide a framework and targets for disrupting pest adaptability by targeting critical symbiont metabolic nodes, positioning microbiome-mediated detoxification as a universal vulnerability in resistant pests.

## Introduction

The fall armyworm (FAW), *S. frugiperda*, has emerged as a globally significant agricultural pest, infesting over 80 crop species and incurring severe economic losses [1]. Recognized by the Food and Agriculture Organization (FAO) as a priority pest for surveillance and mitigation, FAW poses a formidable challenge due to its exceptionally high reproductive rate, near-irreversible establishment once introduced, rapid global spread, and alarming adaptability, coupled with a capacity to evolve resistance to 47 insecticides, rendering conventional pest management strategies progressively ineffective [2–4]. Although resistance mechanisms such as detoxification enzyme overexpression (e.g. cytochrome P450s, glutathione *S*-transferases, esterase, and UDP-glucuronosyltransferase) and target-site mutations have been well documented, these host-centric explanations fall short of fully accounting for the rapidity and variability of insecticide resistance observed in field populations [5, 6]. This disconnect highlights a critical gap: nonhost factors, particularly symbiotic microbes, now, increasingly recognized as key mediators of insect adaptation and invasion, have been underappreciated in this context [7]. Several recent studies have shed light on the role of symbiotic bacteria in shaping the host’s response to insecticides, thereby suggesting that detoxification mechanisms dependent on symbionts (rather than the host itself) merit greater attention.

Emerging research demonstrates that insect-associated microbiota can enhance host adaptation either through direct xenobiotic degradation or modulation of host metabolic pathways [8, 9]. Beyond direct metabolic interactions, microbial provision of essential nutrients may also modulate the host’s detoxification plasticity. Yet, the trophic roles of symbionts in mediating detoxification, particularly via nutritional supplementation, remain unclear. Tryptophan, an essential amino acid that animals cannot synthesize de novo, presents a critical nexus [10, 11]. For the FAW, this nutritional constraint arises from both endogenous biosynthesis incapacity and the inherently low tryptophan content of maize, their primary global host plant [12, 13]. Consequently, microbial-derived tryptophan likely sustains FAW physiology and development, analogous to systems such as gut symbiont *E. casseliflavus* in *Bombyx mori* and endosymbiont *Buchnera* in *Acyrthosiphon pisum*, where symbionts provide tryptophan via conserved biosynthetic pathways [14, 15]. Though tryptophan is the least abundant proteinogenic amino acid in animal tissues [16], it serves dual roles: as a protein building block and as a precursor for a range of bioactive metabolites (e.g. serotonin, kynurenines, and indoles) [17, 18]. These compounds regulate many physiological processes including insecticide defense [19], longevity [20], pathogen resistance [21], circadian clock activity [22], cognitive functions [23], and zinc storage [24]. Despite this framework, the identity of tryptophan-provisioning symbionts in FAWs and their mechanistic contributions to host adaptation, particularly in the context of insecticide resilience, remain unresolved.

Building on our previous identification of *E. casseliflavus* EMBL-3, a gut symbiont capable of insecticide degradation in FAW [25] this study demonstrates that EMBL-3-mediated tryptophan production, rather than its insecticide biodegradative activity, constitutes the primary driver of EMBL-3-mediated FAW survivorship under insecticide stress. Integrating multi-omics, isotope tracing, and CRISPR/Cas9, we dissect a hierarchical symbiont-host detoxification cascade: EMBL-3-generated tryptophan is metabolized by gut microbes via cross-feeding, an ecological interaction where one organism utilizes metabolic products or by-products produced by another organism, into indoleacetic acid (IAA), which activates the host aryl hydrocarbon receptor (AHR) to upregulate the key detoxification enzyme UDP-glucuronosyltransferase 2 (UGT2). This division of labor, tryptophan synthesis by EMBL-3 and conversion to bioactive IAA by consortia, reveals insecticide resistance as an emergent trait forged through microbial metabolic crosstalk, not host or symbiont action alone. By unmasking this covert mechanism, we redefine the functional axis of pest-symbiont interactions and propose precision strategies targeting microbial metabolic hubs to disrupt adaptive resilience.

## Materials and methods

### Insects rearing

FAWs were collected from maize fields in Nanning, Guangxi, China (108.28°E, 23.16°N) and maintained on an artificial diet (Method S1) under controlled conditions (25 ± 1°C, 65.0% RH, 16:8 L:D photoperiod, Table S1). Antibiotic treatment was administered using the diet-overlay method: 1000 mg/l tetracycline was applied to the diet surface to ensure even coverage of antibiotic solution. Third-instar larvae were transferred to the antibiotic-covered diet for 24 h for pretreatment with antibiotics.

### Insecticide bioassay

For the insecticide bioassay, chlorantraniliprole was initially prepared as a stock solution at a concentration of 1000 mg/l. This solution was then diluted with 0.1% Triton X-100 in distilled water to create a series of five concentration gradients. Each gradient solution was added to a 24-well dish containing 900 μl of artificial diet per well, maintaining a diet-to-insecticide solution ratio of 9:1. After allowing the wells to dry, three instars of FAWs were introduced into the wells, and the mortality rate was assessed after 48 h. The lethal concentration for 50.0% of the population (LC_50_) of chlorantraniliprole for FAWs was calculated based on mortality and concentration data. Once the LC_50_ was established, the differences in insecticide susceptibility among various treatments were analyzed using survival curves. In summary, chlorantraniliprole was prepared at the LC_50_ concentration (i.e. 20 mg/l) and administered to FAWs using the aforementioned method. Mortality rates were recorded every 12 h, if an insect could not move normally, it was considered dead, and survival curves were plotted accordingly. The experimental design for assessing the direct and indirect contribution of EMBL-3 to insecticide detoxification in FAWs using the insecticide bioassay is detailed in Method S2.

### Metabolite extraction of fall armyworms and symbionts

Pre-cultures of symbionts isolated from FAWs, including strains EMBL-1 (NCBI accession no.: CP079802) and EMBL-3 (CNGBdb accession no.: CNS0803865), were prepared in 10 ml of LB medium and grown to an OD_600_ of 0.3. Cells were harvested by centrifugation (3000 × g, 4°C, 2 min) and washed with phosphate-buffered saline (PBS). The bacterial pellet was then resuspended in 10 ml of modified M63 medium (glucose 2 g L^−1^, casamino acids 2 g L^−1^, [NH_4_]_2_SO_4_ 2 g L^−1^, KH_2_PO_4_ 13.6 g L^−1^, MgSO_4_.7H_2_O 1 g L^−1^ and FeSO_4_.7H_2_O 0.5 g L^−1^) [15]. Cultures were incubated statically at 37°C for 24 h. After incubation, the medium was centrifuged, filter-sterilized through a 0.22 μm membrane, and 100 μl of the filtrate was mixed with 900 μl of artificial diet in 24-well plates. This mixture was fed to antibiotic-pretreated FAWs. A chlorantraniliprole bioassay was conducted 48 h later.

Three treatments were employed for metabolome analysis: Control (normal diet FAWs), Antibiotic (antibiotic-treated FAWs), and EMBL-3 (FAWs pretreated with antibiotics and then infected with EMBL-3). Each treatment included four biological replicates, with each replicate consisting of five FAWs. After treatment, the FAWs were collected in 2 ml tubes. Following quick freezing with liquid nitrogen, the ground tissue samples were re-suspended in methanol, and the metabolites were fully dissolved by vortexing. The samples were centrifuged at 20 000 × *g* at 4°C for 20 min, dried with a nitrogen blower, and then re-dissolved in 80.0% methanol. Non-targeted metabolome analysis was conducted using an LC–MS system comprising an Agilent 1290 Infinity II UHPLC system tandem with Agilent6545 Q-TOF/MS (Agilent). Extracted metabolites were dissolved in 100 μl of 80% methanol for analysis. Chromatographic separation was achieved on an ACQUITY UPLC BEH Amide column (100 mm × 2.1 mm, 1.7 μm). The mobile phase consisted of two components: (A) 15 mM ammonium acetate and 0.3% NH_3_·H_2_O in water; and (B) 15 mM ammonium acetate and 0.3% NH_3_·H_2_O in 9:1 acetonitrile/water. The flow rate was 0.3 ml /min. The column was eluted with 95% mobile phase B for 1 min, followed by a linear gradient to 50% mobile-phase B over 8 min, held at 50% for 3 min, a linear gradient to 95% mobile phase B over 0.5 min, then 1.5 min at 95% mobile-phase B. The sample volume injected was 5 μl. Mass spectrometry (MS) data were acquired using electrospray ionization in both positive and negative ion modes over a mass-to-charge ratio (m/z) range of 50–1250. The other MS settings included a sheath gas temperature of 350°C, a sheath gas flow rate of 11 l/min, a capillary voltage (VCap) of 4000 V, a nozzle voltage of 1000 V, a gas temperature of 350°C, a nebulizer gas pressure of 30 psi, a drying gas flow rate of 8 l/min, a fragmentor voltage of 110 V, and a skimmer voltage of 65 V. Raw data were processed using Profinder 10.0 (Agilent) for peak detection, alignment, and integration. Based on metabolite abundances, clustering analysis was performed using the MetaboAnalyst online tool (https://www.metaboanalyst.ca/), along with compound variance analysis and KEGG enrichment analysis. [26]

### Identification of biosynthetic gene clusters of tryptophan

The reference genome sequences of FAWs (GCF_011064685.2) and their symbionts EMBL-1 (CP079802), and EMBL-3 (CNS0803865) were downloaded from the National Center for Biotechnology Information (NCBI) database on 20 September in 2023 and gene function prediction was performed using eggNOG-mapper (http://eggnog-mapper.embl.de/ [27]). Then the tryptophan synthesis gene cluster was identified, including *trpA*, *trpB*, *trpC*, *trpD*, *trpE*, *trpF*, *aroA*, *aroB*, *aroC*, *aroD*, *aroE*, *aroF*, *aroG*, *aroH*, *aroK*, and *aroL*.

### Isotope labeling of cells and tryptophan labeling detection

The cells of strain EMBL-3 were cultured in a modified M63 medium, as described above, but containing 2 g L^−1^ 99.0% ^13^C_6_-glucose (Cambridge Isotope Laboratories, Inc.) instead of ^12^C_6_-glucose as the sole carbon source. Bacterial cells were subcultured twice using the same medium. EMBL-3 samples were collected daily, and the ^13^C-labeling status of the harvested bacterial cells was determined based on analysis with a Nu Evolution Gas Isotope Mass Spectrometry, (Nu Instruments., Ltd.) with: Emission:2.6 mA, Trap: 750 V, Block Temp:136°C, Source HT:6 kV, Faraday and IC Collector, based on total carbon content relative to unlabeled controls at the Shanghai Institute of Chemical Industry (Fig. S1A).

To eliminate the interference of diet tryptophan, we developed an artificial diet devoid of any amino acids to assess the symbiont-derived small molecule metabolites on host phenotype. This diet consisted of 300 g L^−1^ of soluble starch (Sigma-Aldrich Corp. MO, USA), 16 g L^−1^ of agar (Sigma-Aldrich Corp. MO, USA), 2 g L^−1^ of [NH_4_]_2_SO_4_ (Sinopharm Group Co., Ltd.,), and 2 g L^−1^ of ^13^C_6_-glucose (Cambridge Isotope Laboratories, USA.). The diet was used to feed ^13^C-labeled EMBL-3 to the axenic FAWs (Method S3), and a FAW without EMBL-3 as control group. These insects were allowed to consume the same above nontryptophan artificial diet for 5 days. After this period, the FAWs were sampled and ground to measure the ratio of ^13^C-labeled tryptophan to total tryptophan using IRMS.

### Effects of tryptophan and its metabolites on chlorantraniliprole detoxification in fall armyworms

Tryptophan (100 mg L^−1^) and its metabolites (100 mg L^−1^) were dissolved in acetone (IAA and 5-hydroxyindoleacetic acid: 5-HIAA) or water (tryptophan and kynurenine), respectively. The diet-overlay method was employed to administer tryptophan and its metabolites to sterile FAWs (Method S4), as described above. After 48 h, the treated FAWs were used for chlorantraniliprole bioassays. FAWs fed only acetone/water served as the control group.

The tryptophan-targeted metabolome analysis of FAWs was used to determine the concentrations of different tryptophan metabolites, the detailed information see Method S4.

### DNA extraction, metagenomic sequencing, and data processing

Total genomic DNA from 16 samples of FAWs (Method S1) collecting at eight provinces of China were extracted using the OMEGA Mag-Bind Soil DNA Kit (M5635–02) (Omega Bio-Tek, Norcross, GA, USA). The genomic DNA was sent to the Personal Biotechnology Co., Ltd. (Shanghai, China) for metagenomic sequencing on the NovaSeq 6000 System (Illumina) with a paired-end 150 bp strategy. After quality control and low-quality sequence filtering, the clean data were then analyzed using the MetaWRAP pipeline (v1.3.0). High-quality draft genomes were then extracted from the previously generated metagenome-assembled genomes (MAGs) using the bin refinement module, and the bin reassembly module was applied to improve the MAG quality by enhancing the completeness and reducing contamination. Only MAGs with an overall quality ≥50.0% (completeness -5 × contamination) were retained for downstream analysis [28]. Open reading frames (ORFs) were predicted for each high-quality MAGs using Prodigal (v2.6.3) [29]. For details, see Method S5.

### Indoleacetic acid production of tryptophan metabolizing symbionts

To measure IAA production ability, we first enriched, isolated, and identified tryptophan-metabolizing symbionts from the FAW gut, then performed genomic sequencing to identify metabolic pathways (Method S6). To measure IAA concentration, 10.0% of resting above symbionts isolate cells (OD_600_ = 1.0) was added to a 20 ml minimal salt liquid medium (MSM) containing tryptophan (1 mg/l). It was incubated at 30°C and 220 rpm for 24 h. The noninoculated culture served as a control. The residual concentration of IAA and tryptophan was extracted by ethyl acetate and measured by HPLC (Method S7). For cross-feeding analysis, we first cultured EMBL-3 in LB medium for 24 h. The cells were then removed by centrifugation at 3000 rpm and 4°C, and the resulting supernatant (containing EMBL-3-derived tryptophan) was filtered through a 0.22 μm membrane to eliminate residual cells. This filtered medium was subsequently used to culture EMBL-SM for 24 h. Afterward, the EMBL-SM culture supernatant was collected, extracted with ethyl acetate to separate tryptophan an IAA, and analyzed by HPLC (Methods S7). LB medium without prior EMBL-3 culture served as the control.

### Aryl hydrocarbon receptor antagonist treatment

The topical treatment on the back of FAWs with AhR antagonist was performed as previously described with modifications [23]. The AhR antagonist CH223191 (Sigma-Aldrich) was resuspended in dimethyl sulfoxide (DMSO; Sigma-Aldrich) at a concentration of 10 mg/ ml, and dilution with acetone to 1 mg/ ml for used. The FAWs were firstly transferred to an ice box to make them suspended animation. For the AhR^−^ group, 1 μl of above solution was applied to the center of the dorsal (segment III-IV) of FAWs. Insects treated with 1 μl of acetone on the center of the dorsal (segment III-IV) served as control (AhR^+^ group). FAWs were held immobile for 30 s after treatment to allow the solvent to penetrate the cuticle in ice box. Treated FAWs were kept in an incubator (25°C, RH 65.0%) for 24 h. Then the insecticide susceptibility and gene expression of treated FAWs were measured using insecticide bioassay and qRT-PCR (method S8), and the primer sequences are shown in Table S2.

### Molecular docking of AhR and IAA

The initial structures of AhR of FAW were predicted with Alphafold3 (https://golgi.sandbox.google.com/) [30], and then employed as search models. The structure of IAA was generated using ChemDraw Professional v17.0 and nonpolar H atoms were merged onto the ligand and the target using AutoDock Tools before performing the docking. The catalytic pocket analysis was carried out by ProteinsPlus Server (https://proteins.plus/) [31]. Molecular docking between IAA and putative AhR structures was carried out using semi-flexible docking approaches in AutoDock Vina [32]. The potential docking poses were further analyzed using PyMol [33].

### Gene screening regulated by the aryl hydrocarbon receptor pathway

The gene screening using transcriptome sequencing (Method S9) and real-time fluorescence quantitative PCR (qRT- PCR) with control, antibiotics, EMBL-3 fed, insecticide exposure, and tryptophan fed FAWs.

### sgRNA design, synthesis, and gene knock out with CRISPR/Cas9 technology

To clarify the role of *UGT2* in host insecticide detoxification, we employed the CRISPR/Cas9 system to knockout the detoxifying gene of FAWs, aiming to induce base insertions or deletions that lead to premature termination of the encoded proteins. A pair of sgRNA against *UGT2* were designed using the sgRNAcas9 (V2.0) design tool [34]. The sgRNA target sequences were checked in a search of the FAW genome using the sgRNAcas9 design tool, and no potential off-target sites were identified (Table S3). A PCR-based approach was used to synthesis sgRNAs following the manufacturer’s instructions (GeneArt Precision gRNA Synthesis Kit, Thermo Fisher Scientific, USA). The Cas9 protein (GeneArt Platinum Cas9 Nuclease) was purchased from Thermo Fisher Scientific (Shanghai, China).

Freshly laid eggs derived from the Nanning strains (within 2 h after oviposition) were washed using distilled water and stuck on a microscope slide with double-side adhesive tape. 1.5 nl of a mixture of two sgRNAs (150 ng/μl) and Cas9 protein (50 ng/μl) was injected into each embryo using Nanoject III (Drummond, Broomall, USA). The injected eggs were incubated at 25°C and 65.0% RH for hatching. After eclosion (generation 1: G1), the hind legs of the adult FAWs were removed for rapid DNA testing. Specifically, the single hind legs were placed in a 1.5 ml centrifuge tube with two steel balls of ~2 mm diameter (after autoclaving), and 25 μl solution (PBS and protease K with a ratio of 23: 2) were added, homogenized at 60 Hz for 2 min, microcentrifuge at 2000 × *g* for 2 min, and supernatant was taken the template for PCR detection of mutation. Primers were shown in Table S4. Once the editing of the fragment is confirmed, the female or male will mate with a wild bear of the corresponding sex to lay eggs (G2), self-fertilize the offspring to produce a new offspring G3, which is examined by the above method, retaining both pure and mutated individuals.

### Statistical analysis

The LC_50_ of FAWs to insecticide was calculated by Polo Plus (ProbitLogitAnalysis, Le-OraSoftware) and the survival curve analysis was performed by GraphPad Prism (Version 6.0). The visualization of gene clusters is performed using gggenes (version 0.5.1) and ggplot2 (version 3.4.1) package in R (version 4.2.3). principal coordinate analysis (PCoA) was performed based on distance matrices using the cmdscale function in R. The correlation between transcript levels and metabolite abundances were performed using Procrustes analysis in R package vegan (v2.5). All results from R were visualized and plotted using ggplot2 (3.3.3). GraphPad Prism version 6.0 and Omicstudio were used for statistical analyses and data plotting. Statistical comparisons were performed with Student’s *t*-test, and survival curve was analyzed by the log-rank (Mantel–Cox) test. The significance level was set at ^*^P < .05 (and different letters) and ^**^P < .01.

## Results

### Gut symbiont metabolism exacerbates insecticide resistance of fall armyworms

**Figure 1:**
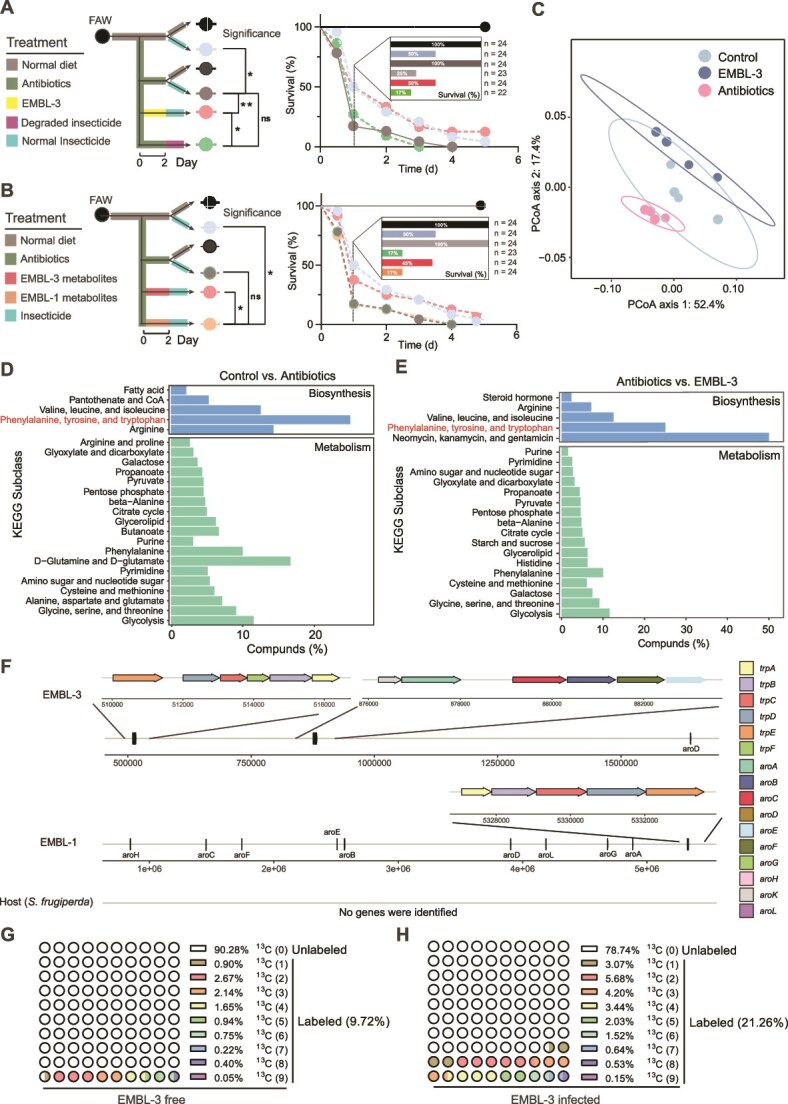
EMBL-3 synthesis and supplies tryptophan to fall armyworms (FAWs). A: Susceptibility of EMBL-3-infected and uninfected FAWs to chlorantraniliprole degraded (48 h) or nondegraded by EMBL-3. B: Effect of EMBL-3 and EMBL-1 metabolites on FAW susceptibility to insecticides. C: Principal coordinate analysis (PCoA) of metabolomes from wild-type, antibiotic-treated, and EMBL-3-infected FAWs. D and E: KEGG enrichment analysis of differentially abundant metabolites between control vs. antibiotic-treated (D) and antibiotic-treated vs. EMBL-3-infected (E) FAWs. F: Tryptophan synthesis potential of FAWs, EMBL-1, and EMBL-3. G and H: Percentage of ^13^C-labeled tryptophan in uninfected (G) and EMBL-3-infected (H) FAWs. 13C(n), “n” reflects the number of labeling ^13^C among nine carbon atoms of tryptophan. Survival curve significance was determined by the log-rank (Mantel–Cox) test (*n =* 24), where “^*^” and “^**^” indicate significant differences *P* < .05 and *P* < .01, respectively, ns, not significant.

To address whether microbial symbiont EMBL-3 facilitates host insecticide resistance through mechanisms other than biodegradation, we examined the susceptibility of FAWs under two conditions: (i) FAWs without EMBL-3 exposed to 20 mg/l chlorantraniliprole predegraded by EMBL-3 in vitro for 48 h, and (2) FAWs infected with EMBL-3 exposed to the same initial concentration (20 mg/l) of chlorantraniliprole (Method S2). Strikingly, we found that the survival rate of EMBL-3-infected FAWs exposed to 20 mg/l chlorantraniliprole was significantly higher than that of the EMBL-3-free FAWs exposed to the predegraded chlorantraniliprole (*P* = .01, Fig. 1A). This finding suggests that the detoxification of chlorantraniliprole by EMBL-3 through biodegradation alone cannot fully explain the observed differences in insecticide resistance. If biodegradation were the sole mechanism, the survival rates of FAWs exposed to predegraded insecticide in vitro should have been comparable to those of EMBL-3-infected FAWs exposed to the initial concentration (20 mg/l) of chlorantraniliprole (Fig. 1A).

We hypothesized that EMBL-3 confers insecticide resistance through an additional mechanism, likely mediated by microbe-host interactions. To test this hypothesis, we evaluated the effects of EMBL-3’s fermented extracts on enhancing insecticide resistance of FAWs, alongside a control group using metabolites from EMBL-1, an intestinal bacterium that does not enhance host insecticide resistance. The results showed that only EMBL-3 metabolites, but not EMBL-1 metabolites significantly increased host insecticide resistance by 28.0% (EMBL-3 vs. antibiotics, *P* = .038; EMBL-1 vs. antibiotics, *P* = 0.77, Fig. 1B). This finding further supports our hypothesis that EMBL-3 enhances host resistance through its metabolic products. To further identify the metabolites involved, we conducted metabolomic analyses and compared the metabolic profiles of FAWs under different conditions. After antibiotics treatment, the bacterial content was significantly decreased, whereas EMBL-3 content was increased after EMBL-3 feeding (Fig. S2). Both antibiotics treatment and EMBL-3 infection significantly altered metabolites associated with biosynthesis and metabolism of FAWs (*P* < .01, Fig. 1C–E). The differentially abundant metabolites in both treatments were enriched in the phenylalanine, tyrosine, and tryptophan biosynthesis pathways, with these pathways showing the highest representation among all KEGG pathways (Fig. 1D and E). Antibiotic treatment (which suppress microbiota metabolism) significantly reduced host amino acid content (*P* = .016), EMBL-3 infection increased it (*P* = .007, Fig. S3A), suggesting that EMBL-3 may primarily supply aromatic amino acids to its host. Genomic sequence annotation analyses of FAWs, EMBL-1, and EMBL-3 revealed that only EMBL-3 possesses a complete tryptophan biosynthesis pathway, whereas the host completely lacks genes required for tryptophan synthesis. In contrast, EMBL-1 lacks the *trpF* gene, rendering its tryptophan pathway incomplete (Fig. 1F). Furthermore, the absence of *tyrB* and *phaA* in EMBL-3’s genome indicates that its ability to synthesize phenylalanine and tyrosine is impaired (Fig. S3B). These findings strongly suggest that EMBL-3 can act as a tryptophan provider for its host.

To confirm the contribution of EMBL-3 in the supply of tryptophan for FAWshost, we then used isotope labeling to trace tryptophan production by EMBL-3 and its utilization by the host. After culturing EMBL-3 with ^13^C-labeled glucose for over two days, more than 75.0% of the carbon atoms in EMBL-3 were replaced with ^13^C-labeled ones (Fig. S1B). When labeled EMBL-3 cells were fed to FAWs reared on a ^13^C glucose diet, EMBL-3-infected FAWs contained 21.3% labeled tryptophan, compared to only 9.7% in EMBL-3-free FAWs (Fig. 1G and H). Our results revealed that EMBL-3 supplied 11.54% of the tryptophan used by FAWs within only five days. Collectively, these results demonstrate that strain EMBL-3 enhances host insecticide resistance through a metabolite-mediated mechanism beyond direct insecticide biodegradation, with tryptophan being the most likely contributing candidate.

### Symbiont-derived tryptophan metabolites promote insecticide resistance of fall armyworms

**Figure 2:**
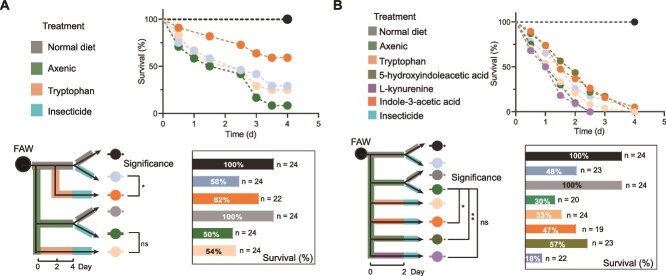
Symbiont-derived tryptophan metabolites from EMBL-3 mediate FAW insecticide resistance. A: Effect of tryptophan on insecticide resistance in wild-type and axenic FAWs. B: Effect of tryptophan metabolites on insecticide resistance in axenic FAWs. Survival curve significance was determined by the log-rank (Mantel–Cox) test, where “^*^” and “^**^” indicate significant differences *P* < .05 and *P* < .01, respectively, ns, not significant.

To determine whether EMBL-3-derived tryptophan contributes to the insecticide resistance of FAWs, we first established an axenic (germ-free) FAW strain as a model organism to eliminate the influence of the host microbiota (Fig. S4A, Method S3). Successful asepsis was confirmed by a significant reduction (by 631.6-fold) in bacterial abundance (Fig. S4B and C). The EMBL-3 content was also increased when axenic FAWs were treated with EMBL-3 (Fig. S4D) We also found that gut microbiota plays a role in FAW development and reproduction but is not essential for basic larval growth (Fig. S5). Next, we supplemented both wild-type (WT) and axenic FAWs with tryptophan and assessed their survival rates under chlorantraniliprole exposure. Tryptophan supplementation significantly increased the survival rate of WT FAWs (by 24.0%) compared to those on a normal diet (*P* = .034), whereas axenic FAWs showed no significant change in survival when fed tryptophan (*P* = .25, Fig. 2A). This finding demonstrated that the promotion of insecticide resistance in FAWs by EMBL-3-derived tryptophan is dependent on the presence of the host microbiota.

Beyond its role in protein synthesis, tryptophan is often metabolized by microorganisms into bioactive derivatives, such as IAA, which can function as ligands for specific receptors. To identify the specific tryptophan metabolites responsible for enhancing insecticide resistance, we investigated three key tryptophan metabolites (i.e. 5-hydroxyindoleacetic acid (5-HIAA), L-kynurenine (Kyn), and IAA), representing distinct metabolic pathways. Feeding experiments revealed that supplementation with IAA (*P* = .019) and 5-HIAA (*P* = .008) significantly increased the insecticide resistance of FAWs to chlorantraniliprole, whereas Kyn (*P* = .66) had no such an effect (Fig. 2B). These results suggest that microbiota-dependent tryptophan metabolites, particularly IAA and 5-HIAA, play a critical role in EMBL-3-mediated promotion of insecticide resistance in FAWs.

### Nondietary IAA plays a key role in enhancing insecticide resistance in FAWs

**Figure 3:**
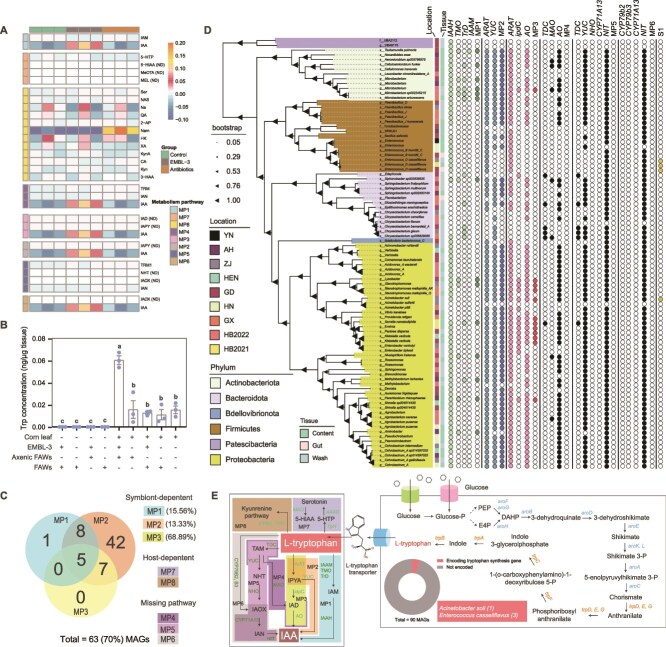
Targeted metabolomics and metagenomics reveal the abundance of tryptophan derivative and associated metabolic pathways in FAW symbionts. A: Relative abundance of tryptophan metabolites across eight metabolic pathways in control, antibiotic-treated, and EMBL-3-infected FAWs. B: Tryptophan content in fasting, maize-fed, and EMBL-3-infected FAWs. Data are mean ± SEM. Significance was determined by one-way ANOVA with multiple comparisons; different lowercase letters indicate significant differences (*P* < .05, *n =* 3). C: Number and proportion of MAGs with potential tryptophan metabolic pathways. D: Maximum-likelihood phylogenetic tree of 90 MAGs from FAW metagenomic data, annotated with genes related to tryptophan synthesis and metabolism. The tree was constructed using GTDB-Tk based on 53 bacterial single-copy genes. E: Tryptophan synthesis and metabolism pathways mapped using the KEGG database. Pathways include host-dependent kynurenine and serotonin pathways and six microbe-dependent IAA production pathways. MP1 to MP9: tryptophan metabolism pathway1 to 9; IAM: Indole-3-Acetamide; IAA: Indole acetic acid; 5-HTP: 5-Hydroxytryptophan; 5-HIAA: 5-Hydroxyindoleacetic acid; MeOTA: 7-Methoxytacrine; MEL: Melationin; Ser: Serotonin; NAS: N-acetyserotnin; QA: Quinolinic acid; 2-AP: 2-aminophenol; Nam: Nicotinamide; HK: 3-Hydroxy-DL-kynurenine; XA: Xanthurenic acid; KynA: Kynurenic acid; CA: Cinnabarinic acid; Kyn: Kynurenine; 3-HAA: 3-hydroxyanthranilic acid; TRM: Tryptamine; IAN: 3-Indoleacetonitrile; IAD: Indole-3-acetaldehyde; IPYA: Indole-3-pyruvate; NHT: N-Hydroxytryptamine; IAOX: Indole-3-acetaldoxime; KYNU: Kynureninase; TDO: Tryptophan 2,3-dioxygenase; MAO: Monoamine Oxydase; AAAD: Aromatic amino acid decarboxylase; TpH: Tryptophan hydroxylase; TDC: Tryptophan decarboxylase; YUC: Flavin-dependent monooxygenase; NHO: Nitrate reductase NIT: Nitrilase; ArAT: Aromatic amino acid aminotransferase; idpC: Indolepyruvate decarboxylase; AO: Aldehyde oxidase; IAAM: Tryptophan monooxygenase; IAAH: indole acetimide hydrolase; TMO: Tryptophan 2-monooxygenase; TrD: Tryptophan decarboxylase; IAAH: Indole acetimide hydrolase; YN: Yunnan; AH: Anhui; ZJ: Zhejiang; HEN: Henan; GD: Guangdong; HN: Hainan; GX: Guangxi; HB: Hubei.

To further investigate which tryptophan metabolite (IAA or 5-HIAA) plays a more critical role in EMBL-3’s collaboration with other symbionts to mediate host insecticide resistance, we conducted targeted metabolomic analyses of tryptophan-related metabolites in FAWs under three conditions: EMBL-3-infected, wild-type (control), and antibiotic-treated (1000 mg/l tetracycline). The results revealed significant differences in tryptophan metabolites between the groups (Fig. S6A and B; PLSDA: *R*^2^ = 0.93, *Q*^2^ = 0.62, Intercept *R*^2^ = 0.81, Intercept *Q*^2^ = −1.46). Among the 24 metabolites detected via LC–MS, the majority (72.0%) were elevated in EMBL-3-infected FAWs (Fig. 3A and S6C). However, eight metabolites, including 5-HIAA, previously implicated in host insecticide resistance (Fig. 2B), were undetectable in FAWs. In contrast, IAA levels were significantly higher in EMBL-3-infected FAWs compared to both control and antibiotic-treated groups (Fig. 3A, S6C, and  S6D). IAA and its intermediate indole −3-acetamide (IAM) showed abundance patterns similar to EMBL-3. Some intermediates were undetected, likely due to complete consumption (Fig. 3A and S6A). These findings suggest that IAA, rather than 5-HIAA, is the primary mediator of EMBL-3-induced enhancement of insecticide resistance in FAWs.

To rule out the influence of artificial diets on insect’s tryptophan supply, we analyzed tryptophan levels in FAWs fed on maize leaves or subjected to fasting, with or without EMBL-3 infection. This approach allowed us to better assess the contributions of EMBL-3-derived tryptophan versus dietary sources. The results showed that EMBL-3 infection significantly increased tryptophan levels in FAWs, with the increase attributable to EMBL-3 being approximately threefold greater than that from maize leaves (*P* = .007, Fig. 3B). Fasted both EMBL-3-infected and noninfected FAWs nearly depleted tryptophan, indicating that EMBL-3 relies on nutrients from maize leaves to synthesize tryptophan (Fig. 3B). Furthermore, the tryptophan content in maize leaves was comparable to that in noninfected FAWs, suggesting that maize leaves are a poor source of tryptophan and that EMBL-3 is the primary provider of this amino acid for FAWs (Fig. 3B). In contrast, maize leaves contained high levels of IAA, but this IAA did not play a role in FAWs, as it might be metabolized by gut microbes or not absorbed by the insects, evidenced by comparable IAA levels in FAWs fed on maize leaves and those that were fasted (Fig. S7B). EMBL-3 infection significantly increased IAA levels in FAWs, regardless of whether they consumed maize leaves (Fig. S7B). These results demonstrated that functional IAA in FAWs is primarily derived from symbionts rather than dietary sources.

### Three parallel gut microbiota-dependent pathways convert EMBL-3-derived tryptophan into IAA

To identify the specific pathways and symbionts involved in tryptophan and IAA production, we performed metagenomic sequencing on field-collected FAWs from eight provinces in China (Table S1). From 16 samples, we assembled 90 MAGs spanning six phyla: *Proteobacteria* (47), *Firmicutes* (15), *Bacteroidota* (14), *Actinobacteriota* (11), *Patescibacteria* (2), and *Bdellovibrionota* (1) (Fig. 3D and E). Using a database of tryptophan synthesis and metabolism enzymes from NCBI, we identified MAGs with complete pathways for tryptophan synthesis or metabolism. Only four MAGs assigned to *Acinetobacter soli* (1) and *E. casseliflavus* (3) potentially possessed complete tryptophan synthesis pathways, with *E. casseliflavus* being significantly more abundant (35-fold) than *A. soli* in the gut microbiota (*P* < .001, Fig. 3C, 3D, and  S7). This suggests that tryptophan synthesis in FAWs is highly specialized and dominated by EMBL-3-like symbionts.

Tryptophan can be converted to IAA through six known pathways, three of which are microbiota-dependent (Fig. 3E). For tryptophan metabolism, 14, 62, and 12 MAGs contained complete pathways I, II, and III, respectively, whereas pathways IV, V, and VI were absent (Fig. 3D and E). Among those MAGs, 5 MAGs possessed all three IAA-producing pathways, and 70.0% of MAGs (63 of 90) were capable of metabolizing tryptophan to IAA (Fig. 3D and E), indicating widespread IAA production potential in the FAW gut microbiota. Specifically, 14 MAGs encoded enzymes for converting tryptophan to IAM via tryptophan 2,3-dioxygenase (TDO) and its homologs, with IAA subsequently produced by hydrolysis of IAM via indole acetamide hydrolase (IAAH) (Fig. 3C–E). Pathway I MAGs were primarily from *Actinobacteriota* and *Proteobacteria*, including four MAGs from the genus *Microbacterium* (Fig. 3E). Pathway II, mediated by aromatic amino acid aminotransferase (AraT), which converts tryptophan to indole-3-pyruvate (IPYA), was the most prevalent (Fig. 3C–E). Because host enzymes also contribute to tryptophan metabolism, pathways II and III likely involve collaboration between host and microbiota, explaining the higher prevalence of MAGs with these pathways (Fig. 3C). Pathways II and III frequently co-occurred, though many MAGs with pathway II lacked a complete pathway III (Fig. 3E). In terms of microbiota abundance, *Klebsiella variicola* and *E. casseliflavus* dominated most wild FAW populations, except for HB2021 and HB2022 (Fig. S8). Collectively, these results demonstrate that FAW gut microbiota plays a widespread and critical role in converting EMBL-3-derived tryptophan to IAA through at least three distinct pathways.

### Gut symbiont-dependent tryptophan cross-feeding enhances insecticide resistance in FAWs

To determine whether bacteria encoding tryptophan metabolic pathways I, II, and III can compensate for EMBL-3-mediated insecticide resistance, we isolated three bacterial strains: *Stenotrophomonas* sp. (EMBL-SM), *K. variicola* (EMBL-1), and *Microbacterium* sp. (EMBL-MO). These strains share genus taxonomic classification with the MAGs containing pathways I, II, and III (Fig. S9A, Method S5). We found that co-culturing EMBL-3 with these three symbiotic bacteria significantly enhanced the host’s detoxification ability to insecticides and rescued the tryptophan-mediated detoxification in sterile FAWs (Fig. S9B). Although EMBL-1 alone could not confer insecticide resistance, its co-infection with EMBL-3 significantly increase host resistance, highlighting the synergistic detoxification potential of microbiota cooperation (Fig. S9B).

**Figure 4:**
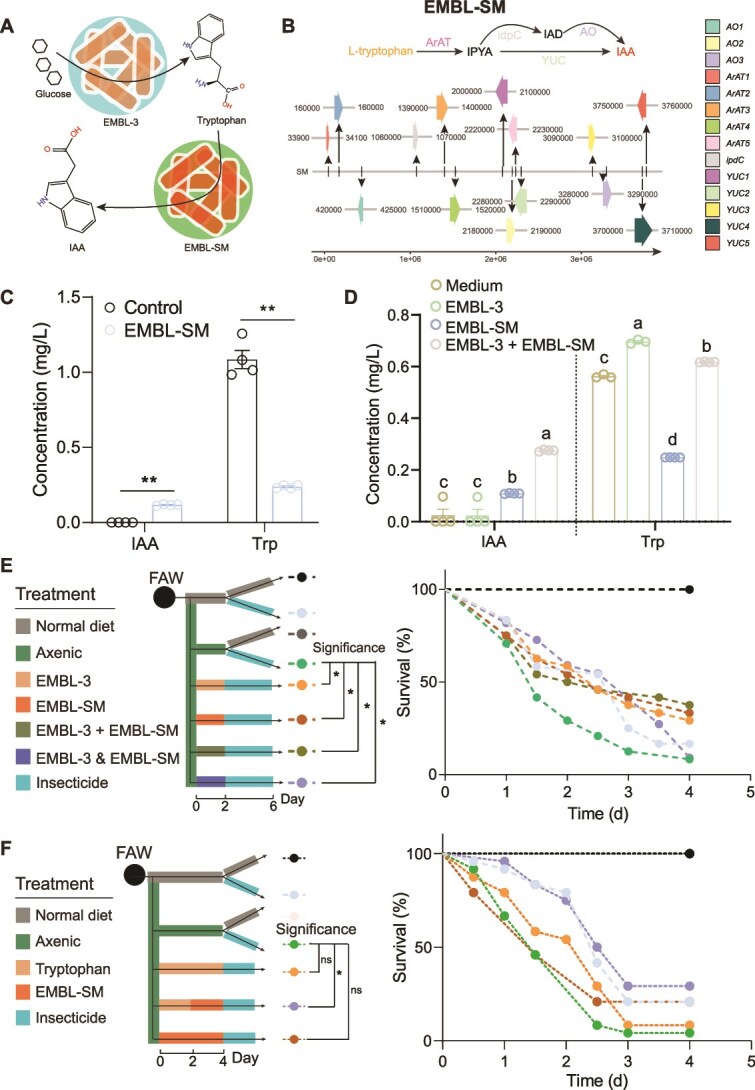
EMBL-3 cross-feeds with EMBL-SM symbionts to produce IAA, mediating FAW insecticide resistance. A: Schematic of EMBL-3 synthesizing tryptophan from glucose and secreting it for conversion to IAA by EMBL-SM via enzymes such as ArAT and YUC. B: Tryptophan metabolism-related genes encoded by the SM genome, primarily involved in pathways II and III. C: Effect of SM on tryptophan and IAA concentrations. Data are mean ± SEM (*n =* 3). ^**^P < .01 (paired Student’s t-test). D: EMBL-SM metabolizes EMBL-3-derived tryptophan to produce IAA. Data are mean ± SEM (*n =* 3). Different lowercase letters indicate significant differences (*P* < .05). E: Effect of SM and EMBL-3 co-infection on FAW insecticide susceptibility. F: Compensation effect of EMBL-SM on tryptophan-mediated insecticide resistance in axenic FAWs. Survival curve significance was determined by the log-rank (Mantel–Cox) test (*n =* 24). Where “^*^” and “^**^” indicate significant differences *P* < .05 and *P* < .01, respectively, ns, not significant.

The whole-genome sequencing of EMBL-SM revealed that this strain encodes all enzymes required for complete tryptophan metabolic pathways II and III, confirming its potential to metabolize tryptophan into IAA (Fig. 4A and B). Culturing EMBL-SM in tryptophan-supplemented medium demonstrated its ability to consume tryptophan and produce IAA (Fig. 4C). Furthermore, co-culturing EMBL-3 with EMBL-SM showed that the latter could utilize tryptophan produced by the former to generate IAA (Fig. 4D). These results demonstrated that EMBL-3 cross-feeds tryptophan to other metabolic symbionts like EMBL-SM, which convert it further into IAA (Fig. 4A). To test whether this cross-feeding behavior could restore tryptophan-dependent insecticide resistance in sterile FAWs, we replaced the microbiota with EMBL-3, EMBL-SM, or a mixture of both (EMBL-3 + EMBL-SM) (Method S10). The result demonstrated that co-infection with EMBL-3 and EMBL-SM resulted in the highest survival rate under insecticide exposure, significantly surpassing that of axenic FAWs (Fig. 4E and F). Additionally, infecting sterile FAWs with EMBL-SM and supplementing tryptophan significantly improved survival rates compared to tryptophan supplementation alone, confirming that IAA, the cross-feeding product of EMBL-3 and EMBL-SM, promotes insecticide resistance in FAWs (Fig. 4E and F). These results revealed that EMBL-3 cross-feeds tryptophan to metabolically complementary, such as EMBL-SM, which subsequently convert it into IAA, thereby enhancing insecticide resistance in FAWs.

### Microbiota-derived IAA promotes insecticide resistance through activating AhR detoxification pathway of FAWs

**Figure 5:**
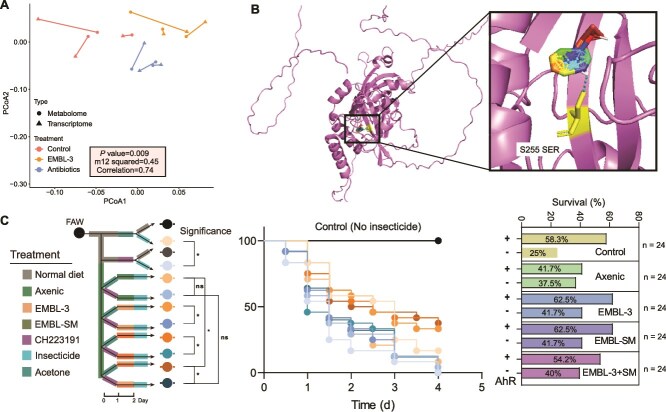
Microbiota-derived IAA activates AhR to promote FAW insecticide resistance. A: Correlation between transcriptomic and metabolomic profiles in FAWs. B: Molecular docking simulation of IAA binding to AhR. C: Effect of AhR inhibition on symbiont-mediated insecticide resistance. Survival curve significance was determined by the log-rank (Mantel–Cox) test (*n =* 24). Where “^*^” and “^**^” indicate significant differences *P* < .05 and *P* < .01, respectively, ns, not significant.

IAA is a known ligand for the AhR, a pathway implicated in detoxification and metabolism in insects [35, 36]. To explore whether microbiota-derived IAA activates the AhR pathway in FAWs, we first performed Procrustes analysis to assess the correlation between metabolomic and transcriptomic data. The results revealed a significant and strong association between metabolite changes and gene expression patterns (Fig. 5A  *P* = .009), suggesting that microbiota-derived metabolites, such as IAA, may reshape host gene expression to enhance detoxification.

We hypothesized that IAA activates the AhR pathway, thereby promoting insecticide resistance in FAWs. To test this hypothesis, we first examined the potential binding of IAA to the FAW AhR using molecular docking. The results indicated that IAA forms hydrogen bonds with the SER255 residue of AhR, with a binding energy of −7.5 kcal/mol, suggesting a potential interaction (Fig. 5B). Although the AhR structure was predicted using AlphaFold3 and requires experimental validation, these findings support the possibility of IAA-AhR binding (Table S5). Next, we treated sterile FAWs with an AhR inhibitor and supplemented them with different bacterial combinations (EMBL-3 and/or EMBL-SM) to assess survival under insecticide exposure. The result showed that AhR inhibition significantly reduced FAW tolerance to chlorantraniliprole, and the insecticide resistance conferred by EMBL-3 and/or EMBL-SM was abolished (*P* < .05, Fig. 5C). This finding demonstrates that AhR activation is essential for the symbiont-mediated enhancement of insecticide resistance.

**Figure 6:**
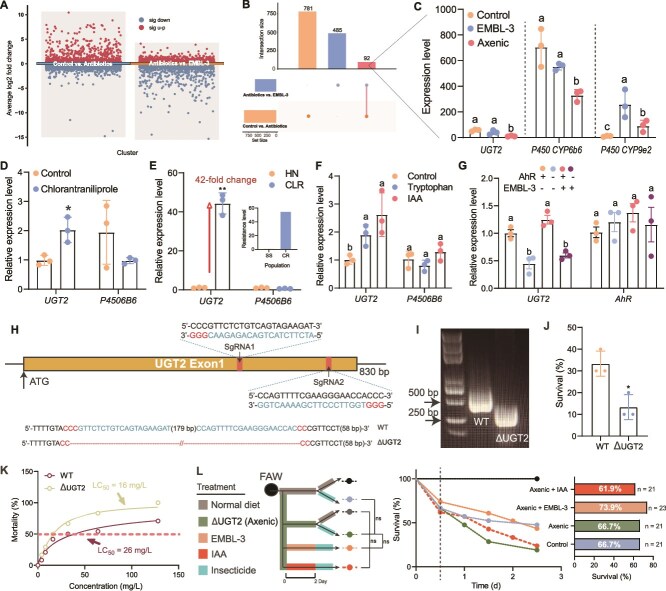
Microbiota-derived IAA activates the AhR-UGT2 pathway to promote FAW insecticide resistance. A: Volcano plots of differentially expressed genes in FAWs under different treatments. B: Overlap of differentially expressed genes between treatments. C: Expression of detoxification genes (*UGT2*, *CYP6b6*, and *CYP9e2*) under different treatments. D: Expression patterns of *UGT2* and *CYP6b6* in response to insecticide exposure. E: Expression of *UGT2* and *CYP6b6* in insecticide-resistant (CLR) and susceptible (HN) FAWs. F: Effect of IAA and tryptophan on *UGT2* and *CYP6b6* expression. G: Effect of AhR inhibition on *UGT2* and *CYP6b6* expression in EMBL-3-infected and uninfected FAWs. H and I: *UGT2* knockout sequence (H) and agarose gel electrophoresis (I) of WT and UGT2-knockout FAWs. J and K: Effect of *UGT2* knockout on FAW survival (J) and LC_50_ (K) under insecticide exposure. L: Effect of IAA and EMBL-3 on insecticide resistance in WT and *UGT2*-knockout FAWs (*n =* 24). Data are mean ± SEM (*n =* 3). ^*^P < .05, ^**^P < .01 (paired Student’s *t*-test). Tryptophan concentration significance was determined by one-way ANOVA with multiple comparisons; different lowercase letters indicate significant differences (*P* < .05, *n =* 3). Survival curve significance was determined by the log-rank (Mantel–Cox) test (*n =* 24). Where “^*^” and “^**^” indicate significant differences *P* < .05 and *P* < .01, respectively, ns, not significant.

To identify the detoxification genes regulated by the AhR pathway, we analyzed transcriptomic profiles of FAWs treated with EMBL-3, control, or antibiotics. A total of 781 and 485 genes were differentially expressed (*P*_adj_ < .05, fold change >1) in the EMBL-3 and control groups, respectively, compared to the antibiotic-treated group, with 92 genes shared between these two treatments (Fig. 6A and B). Among these, three genes from the P450 and UGT families (i.e. *UGT2*, *CYP6b6*, and *CYP9e2*), were highlighted due to their known roles in insecticide resistance (Fig. 6C). Among them, *UGT2* (*P*_adj_ < .01) and *CYP6b6* (*P*_adj_ < .01) were significantly upregulated in both the EMBL-3 and control groups compared to the antibiotic-treated group (Fig. 6C). Quantitative analysis using qRT-PCR revealed that *UGT2* expression exhibited a 42-fold upregulation (*P* = .005) in chlorantraniliprole-resistant FAW strains compared to susceptible counterparts, mirroring the transcriptional induction observed under either insecticide exposure (8.3-fold increase, *P* = .013) or tryptophan dietary supplementation (6.1-fold increase, *P* = .021). Furthermore, *AhR* inhibition significantly reduced *UGT2* expression, regardless of EMBL-3 infection, indicating that AhR regulates *UGT2* independently of symbionts (*P* = .008, Fig. 6G). These new findings in FAWs suggest that *UGT2* is a key target gene regulated by the AhR pathway in response to microbiota-derived IAA.

To directly validate the role of *UGT2* in detoxification, we generated *UGT2* knockout FAWs using CRISPR/Cas9 (Fig. S10). The exon 1 outside the conserved functional domain was identified as the target for knockout, and successful knockout was achieved using a 255 bp sequence containing two sgRNAs (Fig. 6H, 6I, and  S11). The mRNA levels were detected using double primers target on *UGT2* exon 1 and exon 4, respectively, which shown that *UGT2* expression of gene deletion (Fig. S12). *UGT2* deletion significantly reduced FAW survival (by 20.0%) under insecticide exposure (*P* = .013), with the LC_50_ for chlorantraniliprole dropping from 38.61 mg/l to 16.29 mg/l (Fig. 6J and K). The insecticide resistance conferred by IAA and EMBL-3 was abolished in *UGT2* knockout FAWs (Fig. 6L), demonstrating that *UGT2* is essential for fulfilling this resistance mechanism. These results indicate that microbiota-derived IAA promotes insecticide resistance in FAWs by activating the AhR pathway, which upregulates *UGT2* expression to enhance detoxification.

## Discussion

Symbiont-mediated host phenotypic modulation arises not from unilateral action but through intricate metabolic partnerships within microbial consortia [37, 38]. Using FAW as a model, we resolve this trio interaction relationship by unveiling a tripartite EMBL-3–IAA–UGT2 detoxification axis: (i) *E. casseliflavus* EMBL-3 serves as the primary tryptophan supplier via conserved biosynthetic pathways; (ii) cross-feeding symbionts catalyze tryptophan-to-IAA conversion, creating a ligand pool that activates the host AHR signaling cascade; and (iii) AHR-driven transcriptional upregulation of *UGT2*, validated as the key detoxification node, confers insecticide resilience. This cross-feeding between symbionts represents a paradigm shift in resistance evolution, where resistant traits arise from microbial division of labor rather than host genetic adaptation alone. Our work identifies the EMBL-3-IAA metabolic hub as a critical vulnerability point for precision control. These findings position symbiont metabolic networks as high-resolution targets for disrupting the evolutionary arms race between insect pest and insecticide, with potential strategies including designer probiotics, pathway-specific inhibitors, or CRISPR-mediated editing of microbial consortia.

Although dietary intake is traditionally regarded as the primary source of essential amino acids [39], our study confirms an alternative route in FAW: symbiont-derived tryptophan provisioning. EMBL-3 was identified as the keystone tryptophan supplier, a pivotal adaptation for this global invasive pest in maize-based ecosystems where tryptophan is limited. Field surveys confirm EMBL-3’s ubiquity in FAW populations [25, 40], aligning with its unique nutritional role. Metagenomic screening revealed *A. soli* as a secondary tryptophan-producer, yet its negligible abundance (35-fold lower than EMBL-3) underscores EMBL-3’s dominance. Isotope tracing quantified EMBL-3’s metabolic throughput to replenish >20.0% of host tryptophan pools within 6 days, a provisioning efficiency unmatched by dietary or competing microbial sources. This form of *Enterococcus*-mediated tryptophan supplementation appears conserved across lepidoptera, including *B. mori* [15], representing a general microbial strategy to alleviate host nutritional deficits in herbivorous insects. This not only indicates that our findings on FAW are not taxon-specific, but also suggests that exploring variability in this symbiotic trait across Lepidopteran species with distinct feeding ecologies could clarify how host diet shapes symbiont-derived nutrient provisioning. However, the degree of dependence is likely modulated by host diet: species occupying different trophic niches may vary in their reliance on symbionts. Thus, our findings identify EMBL-3 as a promising precision target: modulating its function in the tryptophan, IAA axis could compromise the detoxification flexibility of FAWs, thereby providing a probiotic-guided strategy for mitigating insecticide resistance. This aligns with recent reports of synergistic insecticide-based approaches targeting pest symbiont, among which formulations integrating bacteriophages, small-molecule antibacterial agents (e.g. fungicide), and similar tools are regarded as particularly promising candidates [41, 42].

This study further reveals that EMBL-3 collaborates with phylogenetically diverse gut symbionts to synthesize IAA, linking microbial nutrient exchange to host detoxification in FAW. This cross-kingdom interaction and cross-feeding strategy links microbial nutrient exchange with host detoxification mechanisms, adding complexity to the conventional “one microbe, one metabolite” model [43]. Because IAA acts as a host AhR ligand, emerging evidence suggests that it also influences microbial physiology, such as antibiotic resistance, amino acid metabolism, phage susceptibility, and tolerance to toxic compounds of bacterial cells [44, 45]. This dual role of IAA highlights its significance in shaping both host and microbial physiology [46]. Despite the natural abundance of IAA in plant foliage, FAWs fail to exploit this phyto-pool. This paradox likely arises from two nonmutually exclusive mechanisms: (i) IAA exists primarily as enzymatically cleavable conjugates (e.g. IAA-N-glucosides) requiring hydrolytic activation absent in the FAW gut [46]; and (ii) foregut-resident microbiota rapidly sequester free IAA, preventing its systemic absorption [47]. Experimental evidence confirms this limitation: FAWs only assimilated IAA when it was externally applied at concentrations far exceeding native maize levels (100 μg/g vs. ~0.0001 μg/g in leaf), demonstrating threshold-dependent bioavailability. These findings suggest that IAA-producing bacteria represent both an evolutionary workaround and a mutualistic adaptation beneficial to both insects and plants, compensating for inaccessible dietary IAA in the FAW gut. However, microbial IAA degradation can produce catechol, a compound toxic to insects and plant defense systems. This suggests that IAA production and degradation may reflect a trade-off between plant defense and insect counter-defense mechanisms [47, 48]. Environmental pressures such as tryptophan deficiency may further shape this mutualism between FAWs and its microbiota including EMBL-3. The implications of microbially derived tryptophan and IAA likely extend beyond detoxification, with possible influences on host development and plant-insect interactions. These broader ecological dimensions merit further investigation.

By elucidating the EMBL-3-driven metabolic cascade underlying detoxification, this study advances our understanding of host–microbe interaction networks in shaping host traits like insecticide resistance. By uncovering the unique role of a generalist gut symbiont, *E. casseliflavus* EMBL-3, in enhancing FAW resistance and the underlying mechanism, we propose it as a rational and new probiotic target for symbiont-based insect control (STIC) strategies. Modifying or disrupting this keystone symbiont could reduce FAW resilience and lessen dependence on chemical pesticides, supporting more sustainable pest management, thereby mitigating environmental and human health risks [49]. Our findings suggest that weakening cooperative microbial networks, such as disrupting cross-feeding or inducing exploitative relationships among insect symbionts, could deplete cross-fed or shared metabolites like IAA, diminishing host detoxification capacity or promoting its resistance to insecticides. Although this study only confirms the mechanism by which chlorantraniliprole is affected by this process, the widespread cross-resistance observed in pest populations makes it worthwhile to investigate whether other insecticides are similarly influenced [50].

Ultimately, our findings establish EMBL-3-mediated tryptophan cross-feeding as a linchpin of insecticide resistance in FAWs and provide a high-resolution target for symbiont-based pest control. Because IAA’s role in AhR activation is pivotal, its origins via multisymbiont cross-talk introduces exploitable vulnerabilities. Precision strategies such as bacteriophage engineering, EMBL-3-like bacteria or delivering lytic endolysins to IAA-producing consortia, may decouple this symbiont-driven detoxification cascade [51]. Future efforts should prioritize structural into the IAA-AhR binding and real-time metabolite flux analysis at the plant-insect-microbe interface. Through CRISPR-phage systems, keystone symbionts could be selectively edited or removed and pest resistance be reversed, transforming pest control from chemical intervention to ecological disruption. These approaches, grounded in microbial network vulnerability, could redefine pest management toward predictive, symbiosis-aware agricultural systems, harmonizing agricultural productivity with ecosystem resilience.

## Supplementary Material

SI-R2-clean_wraf237

## Data Availability

The sequencing raw data generated in this study are deposited in the China National GeneBank DataBase (CNP0007137). All source data and datasets can be found in Figshare (https://figshare.com) with 10.6084/m9.figshare.30084226.
