# Supplementary material for: Cooperative microbial metabolism enhances tryptophan-mediated insecticide detoxification in the fall armyworm
Source: ISME J. 2025 Oct 24;19(1):wraf237. doi: 10.1093/ismejo/wraf237 (PMC12624862; doi:10.1093/ismejo/wraf237)
Supplement: SI-R2-clean_wraf237 [file si-r2-clean_wraf237.docx]

**SUPPLEMENTARY MATERIAL for**

# Cooperative Microbial Metabolism Enhances Tryptophan-Mediated Insecticide Detoxification in the Fall Armyworm

*** Correspondence to**

Dr. Feng Ju

Associate Professor

Westlake University

Email: jufeng@westlake.edu.cn

Address: 600 Dunyu Road, Xihu District, Hangzhou 310030, China

Tel.: 571-87963205 (lab), 571-87380995 (office); Fax: 0571-85271986

**Supplementary methods**

**Methods S1: Insect samples and insecticide bioassay**

**Methods S2:** **Determine of direct and indirect contribution of EMBL-3 to insecticide detoxification of FAWs**

**Method S3: Sterile FAW strain generated and EMBL-3 feeding**

## Method S4: Tryptophan-targeted metabolome analysis of FAWs

**Method S5: Enrichment, isolation, and identification of tryptophan metabolizing symbionts and genome sequencing**

## Method S6: Enrichment, isolation, and identification of tryptophan metabolizing symbionts and genome sequencing

**Method S7: IAA and tryptophan concentration measured by HPLC**

**Method S8 Real-time fluorescence quantitative PCR (qRT- PCR)**

## Method S99 Transcriptome sequencing and

**Method S10:** **Detection host insecticide susceptibility after infection of tryptophan anabolic symbionts**

**Supplementary figures and tables**

Table S1 ***Spodoptera frugiperda* field population information used in this study.**

Table S2 Primer sequences for qRT-PCR

Table S3 SgRNA sequences for *UGT2*

Table S4 *UGT2* knockdown detect primer sequences

**Table S5 The scoring of different conformations in the AhR protein structure predicted by AlphaFold3**

**Figure S1 Workflow for ^13^C isotope labeling (a) and ^13^C labeling rate of EMBL-3 cells over time (b)**

**Figure S2 The influence of antibiotics on the total bacterial (a) and EMBL-3 (b) content of FAWs**

**Figure S3 The total amino acid content in FAWs (a) and the aromatic amino acid synthesis gene cluster of EMBL-3 (b)**

**Figure S4 Workflow for generating axenic FAWs**

**Figure S5 Differences in growth, development and reproduction between wild type FAWs (WT) and sterile FAWs (AX)**

**Figure S6 Differences and consistency of tryptophan targeted metabolome in different treatments of FAWs**

**Figure S7 The influence of EMBL-3 abundance on the content of IAA and IAM.**

**Figure S8 Relative abundance of top 10 MAGs among 9 field population of FAWs**

**Figure S9 The influence of EMBL-1 and EMBL-MO on the insecticide susceptibility of FAWs**

**Figure S10 Workflow for CRISPR/Cas9-mediated *UGT2* knockout in FAWs**

**Figure S11 Gene structure (a) and coding protein domain predicted (b) of *UGT2***

**Figure S12 Gene sequence, exon, and primer position information of *UGT2* (a); The mRNA expression level of *UGT2* in wild-type and *UGT2*-knockout strains FAWs**

**.**

Supplementary methods

## Method S1: Insect samples and insecticide bioassay

The wild of FAWs utilized in the metagenomic sequencing experiments were sampled from eight provinces in China, including Yunnan, Henan, Zhejiang, Guangdong, Hainan, Anhui, Hubei and Guangxi in 2022 (Table S1). Larvae were maintained in breathable plastic containers with a continuous supply of artificial diet, while adults were housed in insect cages measuring 30 cm × 30 cm × 30 cm and provided with a 10% honey solution as a nutritional supplement [1]. All insects were reared at a temperature of 25 ± 1 °C and 65 ± 5% relative humidity (RH) with a light: dark (L: D) photoperiod of 16 h:8 h.

## Method S2: Determine of direct and indirect contribution of EMBL-3 to insecticide detoxification of FAWs

The experiments were conducted with five treatment groups, including a normal diet (Control, EMBL-3-free) with or without exposure to chlorantraniliprole, antibiotic treatment (EMBL-3-free) with or without chlorantraniliprole exposure, EMBL-3-infected fall armyworms (FAWs) with chlorantraniliprole exposure, and antibiotic treatment (EMBL-3-free) with EMBL-3 degraded chlorantraniliprole in vitro (Fig. 1b). The hypothesis posits that EMBL-3 degraded chlorantraniliprole is the sole mechanism mediating the detoxification of FAWs. The direct and indirect contributions of EMBL-3 to insecticide detoxification in FAWs were elucidated by comparing the mortality rate of FAWs infected with EMBL-3 at the initial concentration of chlorantraniliprole (20 mg/L) to that of chlorantraniliprole degraded in vitro (~16 mg/L) against EMBL-3 uninfected FAWs.

For fermented extracts on testing insecticide resistance of FAWs, EMBL-3 and EMBL-1 were cultured 24 h in LB medium. The cultures were centrifuged at 4000 rpm for 10 min to pellet the cells, and the resulting supernatants were filtered through 0.22-μm membranes to eliminate residual cellular debris. These filtered supernatants were then incorporated into the diet as supplements containing symbiotic bacterial fermentation products. Two days post-treatment, the effects of the different symbiotic bacterial fermentation products on the insecticidal susceptibility of FAW were evaluated using bioassay methods.

## Method S3: Sterile FAW strain generated and EMBL-3 feeding

To obtain sterile FAWs from wild-type FAWs, we employed an egg surface sterilization method based on a previous study [2]. In detail, the newly produced eggs were individually separated using a small brush and soaked in a 1% sodium hypochlorite solution for 30 s, then transferred to a 75% ethanol solution for an additional 30 s, and subsequently washed three times with sterile water. After the sterilization process, the eggs were placed individually in a 24-well dish containing high-pressure steam-sterilized feed until hatching. The 24-well dish was covered with sterile 200-mesh nylon gauze and a sterile Sealing Tape (Thermo Scientific). Once the sterile larvae hatched, they were continuously fed sterile feed and maintained in sterile conditions until the experiment commenced. The bacterial content of sterile FAWs and wild-type FAWs were then detected using colony counting and real-time fluorescence quantitative PCR (qRT-PCR). Then the EMBL-3 was added in sterile diet to feeding EMBL-3 to FAWs, the EMBL-3 content was detected using specific primer ECHP F (5’- GTTCCCTGGTGCTTATGGCT-3’) and ECHP R (5’- AATTCTGCATCGCAGGGGAA -3’) through qRT-PCR. The insecticide susceptibility, survival, life span and body weight were also analyzed after sterile treatment.

## Method S4: Tryptophan-targeted metabolome analysis of FAWs

Tryptophan-targeted metabolome analysis was conducted using a LC-MS/MS on a Nexera Series LC-40 system coupled with a QTRAP 6500+ mass spectrometer (Sciex, USA) at Majorbio Bio-Pharm Technology Co. Ltd. (Shanghai, China). The 33 standards metabolites in tryptophan pathway were weighed accurately and prepared with 50 % methanol for standard solution. A mixed standard was prepared with taken an appropriate amount above solution. And diluted this mixed standard solution for different concentrations for used. Insect tissue samples was weighed precisely (~50 mg) in a tube, 10 μL internal standard solution (Trp-D5, 4000 ng/mL) and 190 μL of extraction solution (methanol: water=4: 1) were added to the tube. Samples were then homogenated at -10 ℃ by high throughput tissue crusher Wonbio-96c (Shanghai wonbio technology co., LTD) operating at a frequency of 50 Hz for 6 min, then followed by sonicated at 40 kHz for 30 min at 5℃. The samples were placed at -20℃for 30 min. After centrifugation at 13000 g at 4℃ for 15 min, the supernatant was injected into the LC-MS/MS system for analysis. The LC-MS/MS analysis of sample was conducted on a Nexera Series LC-40 system coupled with a QTRAP 6500+ mass spectrometer (Sciex, USA) at Majorbio Bio-Pharm Technology Co. Ltd. (Shanghai, China). Briefly, samples were separated by an ACQUITY UPLC HSS T3 (2.1×150 mm, 1.8 μm) thermostated at 40℃.Separation of the metabolites was achieved at 1 mL/min flow rate with a mobile phase as a gradient consisted of water, containing 0.1% formic acid (solvent A) and 100% acetonitrile in water containing 0.1% formic acid (solvent B). The total chromatographic separation was 18 min. The conditions of the elution gradient were as follows: 0.0-2.5 min, 1%-11% B; 2.5-5.5 min, maintain 11% B; 5.5-6.5 min, 11%-28% B; 6.5-7.5 min, maintain 28% B; 7.5-12.5 min, 28%-50% B; 12.5-13.5 min, 50%-95%B; 13.5-15.5 min, maintain 95%B; 15.5-15.6 min, 95%-1% B; 15.6-18 min, maintain 1% B. During the period of analysis, samples were stored at 4℃.

The mass spectrometric data was collected using a UHPLC coupled to a QTRAP 6500+ mass spectrometer (Sciex, USA) equipped with an electrospray ionization (ESI) source operating in both positive and negative mode. The parameters were set as following: source temperature at 550 ℃; curtain gas (CUR) at 35 psi; CAD gas pressure Medium; both Ion Source Gas1 and Gas2 at 50 psi; ion-spray voltage floating (ISVF) at 5500V /-4500V. Quality control (QC) samples are mixed samples or certain concentrated mixed standard solutions, mainly used to assess the stability of the analytical system. QC samples would be injected at regular intervals (every 10 samples) in order to examine the stability of the analysis, and the RSD of the stability of these targets should be less than 15%. The LC-MS raw data were imported into Sciex software OS. All ion fragments were automatically identified and integrated by using default parameters, besides, all integrations were checked manually. The metabolite concentration of sample was calculated according to linear regression standard curve.

## Method S5: Metagenomic sequencing and data processing

Raw reads of each metagenomic data were filtered to remove adapters and low-quality reads using Trimmomatic (v0.39)[3]. Subsequently, clean reads were *de novo* assembled using the metaspades option in the MetaWRAP pipeline (v1.3.0) with default parameters [4] The assembly generated contigs were binned independently to generate metagenome-assembled genomes (MAGs) using binning software programs MetaBAT2 in the MetaWRAP pipeline with default parameters.

To obtain tryptophan synthesis and metabolism enzyme, we draw a tryptophan synthesis and metabolism ‘circuit board’ according to previous studies and Kyoto Encyclopedia of Genes and Genomes (KEGG, https://www.genome.jp/kegg) database [5, 6]. We then download tryptophan-related protein sequences in NCBI database and using these amnio acid sequences construction database to identify tryptophan synthesis and metabolism protein in FAWs microbiome from MAGs. The tryptophan synthesis and metabolism-like protein were identified by aligning their amnio acid sequences to the databases above using BLASTX at an *E* value ≤ 10^–7^ with at least 50% similarity, more than 150 AA and 70% query coverage [7, 8].

The relative abundance of the MAGs was calculated using quant_bins quant_bins in the MetaWRAP pipeline with default parameters. Briefly, the relative abundance of each MAG was calculated as the number of reads aligning to the MAG normalized by the total number of reads in the sample[4].

## Method S6: Enrichment, isolation, and identification of tryptophan metabolizing symbionts and genome sequencing

To isolate tryptophan-metabolizing bacterial symbionts, the gut of five field FAWs were suspended in 10 mL of PBS and vortexed for 5 min eluting symbiotic bacterial cells. Microcentrifugation (approximately 500 rpm) removes the host’s solid tissue and gut contents. Then the solution was gradient diluted by PBS by 10× from 10^-1^ to 10^-8^ and 50 μL diluent solution was evenly coated on the minimal salt liquid medium plate media with tryptophan as the only carbon and nitrogen source and cultured overnight at 30°C. The next day, different single colonies were selected and added into the 500 μL LB liquid medium without any antibiotics for expanded culture, and plate streak purification and species identification were carried out.

For bacterial symbiont strains identification, a near full-length 16 S rRNA gene sequence was PCR amplified using the universal primers 27 F (5’- AGAGTTTGATCCTGGCTCAG-3’) and 1492 R (5’-GGTTACCTTGTTACGACTT-3’). The 16 S rRNA gene amplicon sequence obtained from Sanger sequencing was deposited in the National Center for Biotechnology Information (NCBI) database and annotated using NCBI’s online Basic Local Alignment Search Tool (BLAST) on 20 September in 2023 based on the BLAST+ 2.13.0.

The genomic DNA of tryptophan metabolizing symbionts (*Stenotrophomonas sp.* SM, *Klebsiella variicola* EMBL-1 and *Microbacterium* sp.) was extracted using the Cetyltrimethyl Ammonium Bromide (CTAB) method. The DNA concentration, quality and integrity were determined using a Qubit Fluorometer (Invitrogen, USA) and a NanoDrop Spectrophotometer (Thermo Scientific, USA). Sequencing libraries were generated using the TruSeq DNA Sample Preparation Kit (Illumina, USA) and the Template Prep Kit (Pacific Biosciences, USA). The genome sequencing was performed by Personal Biotechnology Company (Shanghai, China) by using the Pacific Biosciences and Illumina Novaseq platforms. Data assembly proceeded after removing adapter contamination and filtering the data using AdapterRemoval [9] and SOAPec [10]. The filtered reads were assembled by Unicycler [11]. The complete genomic sequence was annotated by Eggnog-Mapper (<http://eggnog-mapper.embl.de/>) [12]. The genome sketch was created using CGview (<https://proksee.ca/>) with predictions for clustered regularly interspaced short palindromic repeats (CRISPRs), resistance genes, ORFs and GC content calculation [13].

**Method S7: IAA and tryptophan concentration measured by HPLC**

For tryptophan, the Waters H-Class system is equipped with a reversed phase column (BEH C18 1.7 μm 100 mm × 2.1 mm). The mobile phase consisted of HPLC-grade ACN (B) containing 0.2% acetic acid and water (A) containing 0.2% acetic acid at a flow rate of 0.6 mL·min-1. Gradient elution, initial A 100 B 0; 1 min A 100 B 0; 5 min A 95 B 5; 6 min A 90 B 10. The column temperature was 47℃, the sample size was 1.5 μL, and the total running time was 6 min. The wavelength range is 210-400 nm, the selected wavelength is 268 nm, and the peak time is about 2.5 min. For IAA, the Waters H-Class system is equipped with a reversed phase column (BEH C18 1.7 μm 100 mm × 2.1 mm). The mobile phase consisted of CAN (HPLC grade) containing 0.1% acetic acid (HPLC grade) and water containing 0.1% acetic acid at a flow rate of 0.4 mL·min-1. The column temperature was 40 ℃, the sample size was 5 μL, and the total running time was 5 min.

**Method S8 Real-time fluorescence quantitative PCR (qRT- PCR)**

Total RNA of five FAWs with different treatment (Control, antibiotic and EMBL-3) were extracted using RNAiso Plus following the manufacturer’s protocol (TAKARA, DaLian, China). RNA (1 μg) extraction from chlorantraniliprole-exposure, chlorantraniliprole-resistant, susceptible and tryptophan-fed FAWs, were used to synthesize cDNA using the RevertAid First Strand cDNA Synthesis Kit (TAKARA, DaLian, China). Subsequently, to detect the selected gene expression levels according to transcriptome analysis. the cDNA was used as the template for qRT-PCR with 10 μL reactions containing 5 μL of the SoFast EvaGreen Supermix (Bio-Rad, Hercules, CA, USA) and 10 nM of the primers. Housekeeping genes GAPDH and RPL4 were employed as the double references to normalize gene expression levels for FAWs [14]. Details of all primers are shown in Table S2. PCR was run in an qTOWER3 G (Analytik Jena AG, Germany) at the following thermal cycle: initial denaturation at 95 °C for 30 s, followed by 40 cycles of 95 °C for 5 s and 60 °C for 10 s. After the thermal cycles, a melt curve analysis was conducted from 55 to 95 °C. The relative expression levels were calculated based on the 2^−ΔΔCT^ method [15].

## Method S9 Transcriptome sequencing and

Sequencing libraries were generated using the TruSeq RNA Sample Preparation Kit (Illumina, San Diego, CA, USA). Sequencing was conducted on a Hiseq platform (Illumina) by BGI Center Cp. Ltd. Clean reads were obtained by removing raw reads with adaptors, poly-*N*, and having a low quality (< Q20). Gene expression levels were estimated by RSEM software package (http://deweylab. biostat.wisc.edu/rsem). Transcripts were annotated based on the reference genome (NCBIJAKUHG02), and sequences were annotated to the KEGG ORTHOLOGY (KO) database with the KEGG Automatic Annotation Server. Differentially expressed genes were performed using the DESeq2 version 1.28.1 package in R.

**Method S10:** **Detection host insecticide susceptibility after infection of tryptophan anabolic symbionts**

The diet-overlay method was used to construction the tryptophan metabolizing and synthesis symbionts infected strains of FAW. In detail, Axenic FAWs were fed with EMBL-3 for 2 days then transfer insects to new diet containing tryptophan metabolizing symbionts, including SM1, EMBL-1 and *Microbacterium* sp. for 2 days. Synthesis symbionts community was co-culture two symbionts in a 10 mL LB medium overnight, and then centrifuge to discard supernatant. The bacterial cells were washed with sterile PBS twice, then using 2 mL PBS suspensions and fed to sterile larvae trough diet-overlay method. These above treated FAWs were used to insecticide bioassay.

We also chose symbionts SM1 to test the influence of tryptophan on the insecticide susceptibility of Axenic FAWs. After axenic FAWs SM1 colonization using above methods, tryptophan was fed to both axenic and SM1 FAWs for 2 days, then this treated FAWs were used to insecticide bioassay.

**Reference**

1. Zhang Y et al. Symbiont community assembly shaped by insecticide exposure and feedback on insecticide resistance of Spodoptera frugiperda. *Commun Biol* 2024;**7**:1194. https://doi.org/10.1038/s42003-024-06892-1

2. Wu J et al. Axenic and gnotobiotic insect technologies in research on host–microbiota interactions. *Trends Microbiol* 2023;**31**:858–871. https://doi.org/10.1016/j.tim.2023.02.007

3. Bolger AM, Lohse M, Usadel B. Trimmomatic: a flexible trimmer for Illumina sequence data. *Bioinformatics* 2014;**30**:2114–2120. https://doi.org/10.1093/bioinformatics/btu170

4. Uritskiy G V., DiRuggiero J, Taylor J. MetaWRAP—a flexible pipeline for genome-resolved metagenomic data analysis. *Microbiome* 2018;**6**:158. https://doi.org/10.1186/s40168-018-0541-1

5. Agus A, Planchais J, Sokol H. Gut Microbiota Regulation of Tryptophan Metabolism in Health and Disease. *Cell Host Microbe* 2018;**23**:716–724. https://doi.org/10.1016/j.chom.2018.05.003

6. Mano Y, Nemoto K. The pathway of auxin biosynthesis in plants. *J Exp Bot* 2012;**63**:2853–2872. https://doi.org/10.1093/jxb/ers091

7. Zhang Z, Zhang G, Ju F. Using Culture-Enriched Phenotypic Metagenomics for Targeted High-Throughput Monitoring of the Clinically Important Fraction of the β-Lactam Resistome. *Environ Sci Technol* 2022;**56**:11429–11439. https://doi.org/10.1021/acs.est.2c03627

8. Zheng Y et al. Novel order-level lineage of ammonia-oxidizing archaea widespread in marine and terrestrial environments. *ISME J* 2024;**18**:wrad002. https://doi.org/10.1093/ismejo/wrad002

9. Lindgreen S. AdapterRemoval: easy cleaning of next-generation sequencing reads. *BMC Res Notes* 2012;**5**:337. https://doi.org/10.1186/1756-0500-5-337

10. Luo R et al. SOAPdenovo2: an empirically improved memory-efficient short-read de novo assembler. *Gigascience* 2012;**1**:18. https://doi.org/10.1186/2047-217X-1-18

11. Wick RR et al. Unicycler: Resolving bacterial genome assemblies from short and long sequencing reads. *PLoS Comput Biol* 2017;**13**:e1005595. https://doi.org/10.1371/journal.pcbi.1005595

12. Cantalapiedra CP et al. eggNOG-mapper v2: Functional Annotation, Orthology Assignments, and Domain Prediction at the Metagenomic Scale. *Mol Biol Evol* 2021;**38**:5825–5829. https://doi.org/10.1093/molbev/msab293

13. Grant JR, Stothard P. The CGView Server: a comparative genomics tool for circular genomes. *Nucleic Acids Res* 2008;**36**:W181–W184. https://doi.org/10.1093/nar/gkn179

14. Guo Z et al. Insecticide Susceptibility and Mechanism of *Spodoptera frugiperda* on Different Host Plants. *J Agric Food Chem* 2022;**70**:11367–11376. https://doi.org/10.1021/acs.jafc.2c04189

15. Livak KJ, Schmittgen TD. Analysis of Relative Gene Expression Data Using Real-Time Quantitative PCR and the 2−ΔΔCT Method. *Methods* 2001;**25**:402–408. https://doi.org/10.1006/meth.2001.1262

Table S1 ***Spodoptera frugiperda* field population information used in this study.**

| **Population** | **Host plant** | **Province, city** | **Site** | **Date** |
| --- | --- | --- | --- | --- |
| AH | Corn | Anhui, Wuwei | 117.91 °E, 31.15 °N | 2022-08-31 |
| GX | Corn | Guangxi, Nanning | 108.25 °E, 22.85 °N | 2022-09-06 |
| NN | Corn | Guangxi, Nanning | 108.28 °E, 23.16 °N | 2022-09-16 |
| GD | Corn | Guangdong, Zhanjiang | 110.23 °E, 20.73 °N | 2022-08-17 |
| HB2022 | Corn | Hubei, Jingzhou | 112.14 °E, 30.20 °N | 2022-07-20 |
| ZJ | Sorghum | Zhejiang, Dongyang | 120.32 °E, 29.28 °N | 2022-08-08 |
| HB2021 | Corn | Hubei, Jingzhou | 112.14 °E, 30.20 °N | 2021-08 |
| HN | Corn | Hainan, Danzhou | 109.55 °E, 19.59 °N | 2021-08 |
| HEN | Corn | Henan, Xinyang | 114.12 °E, 32.13 °N | 2021-08 |
| YN | Corn | Yunnan, Puer | 100.89 °E, 22.26 °N | 2021-08 |
| HN (CS) | Corn | Hainan, Sanya | 109.12°E, 18.37°N | 2021 |
| HN (CR) | Corn | Obtained from CS after 15 generations of Elimination selection with chlorantraniliprole. | | |

Table S2 Primer sequences for qRT-PCR

| Primers | Sequences (5’-3’) |
| --- | --- |
| *AhR*-F | TCCATGAGAGGCGGTGATTG |
| *AhR*-R | CAATGCGCACAGCAAACAAAA |
| *UGT2*-F1 | GTGGGCAGAGTACCTCGAAC |
| *UGT2*-R1 | GCAGAATGATGGTAGCTGTGG |
| *UGT2*-F2 | TGCACGAACTTGCTCGAAGA |
| *UGT2*-R2 | TGGCTGGTGTTCCTCCTTTG |
| P450 *CYP6B6*-F1 | TCAAGCGTATAGCCCGTGTT |
| P450 *CYP6B6*-R1 | TGTTCTTCTTCTTCGTGTCAAGG |
| *RPL4*-F | CAACAAGAGGGGTTCACGAT |
| *RPL4*-R | GCACGATCAGTTCGGGTATC |
| *GAPDH*-F | CGGTGTCTTCACAACCACAG |
| *GAPDH*-R | TTGACACCAACGACGAACAT |

Table S3 SgRNA sequences for *UGT2*

| Primers | Sequences (5’-3’) |
| --- | --- |
| *Sg1* | **GGGUGGUUCCCUUCGAAAACGUU**UUAGAGCUAGAAAUAGCAAGUUAAAAUAAGGCUAGUCCGUUAUCAACUUGAAAAAGUGGCACCGAGUCGGUGCUUUU |
| *Sg2* | **AUCUUCUACUGACAGAGAACGUUU**UAGAGCUAGAAAUAGCAAGUUAAAAUAAGGCUAGUCCGUUAUCAACUUGAAAAAGUGGCACCGAGUCGGUGCUUUU |

Table S4 *UGT2* knockdown detect primer sequences

| Primers | Sequences (5’-3’) |
| --- | --- |
| *UGT2*-de-F | TACCGCATATGGCACGAACA |
| *UGT2*-de-R | GCGGCTCTGTCAGGAATCTG |

**Table S5 The scoring of different conformations in the AhR protein structure predicted by AlphaFold3.**

| Number | chain_pair_iptm | chain_ptm | ptm | fraction_disordered | ranking_score | pLDDT（Maximum - Minimum） |
| --- | --- | --- | --- | --- | --- | --- |
| 0 | 0.34 | 0.34 | 0.34 | 0.49 | 0.59 | 6.91 - 97.82 |
| 1 | 0.35 | 0.35 | 0.35 | 0.47 | 0.59 | 6.10 - 97.70 |
| 2 | 0.34 | 0.34 | 0.34 | 0.48 | 0.58 | 7.10 - 97.82 |
| 3 | 0.35 | 0.35 | 0.35 | 0.46 | 0.58 | 6.38 - 97.86 |
| 4 | 0.34 | 0.34 | 0.34 | 0.45 | 0.57 | 6.11 - 97.58 |


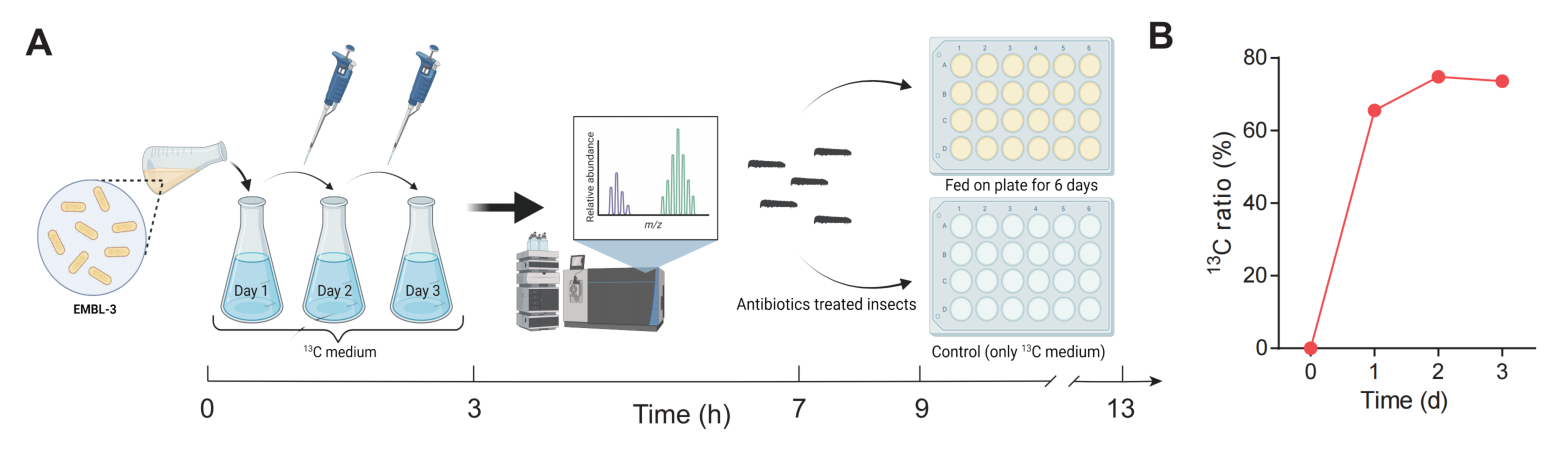


**Figure S1 Workflow for ^13^C isotope labeling (A) and ^13^C labeling rate of EMBL-3 cells over time (B).**


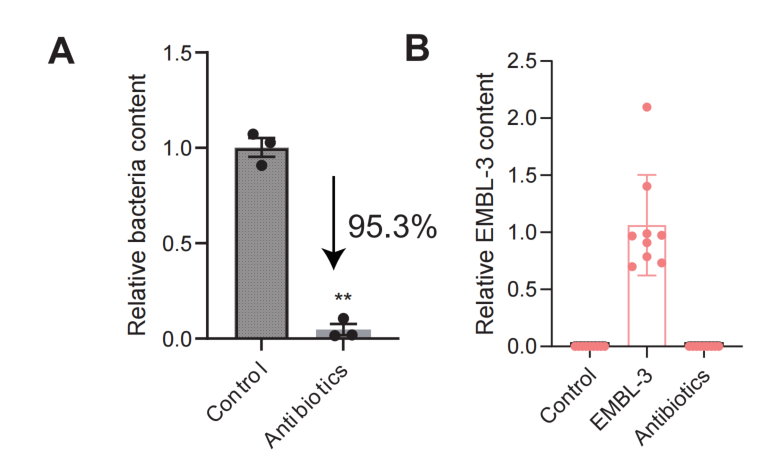


**Figure S2 The influence of antibiotics on the total bacterial (A) and EMBL-3 (B) content of FAWs.**

Note: Data are mean ± SEM


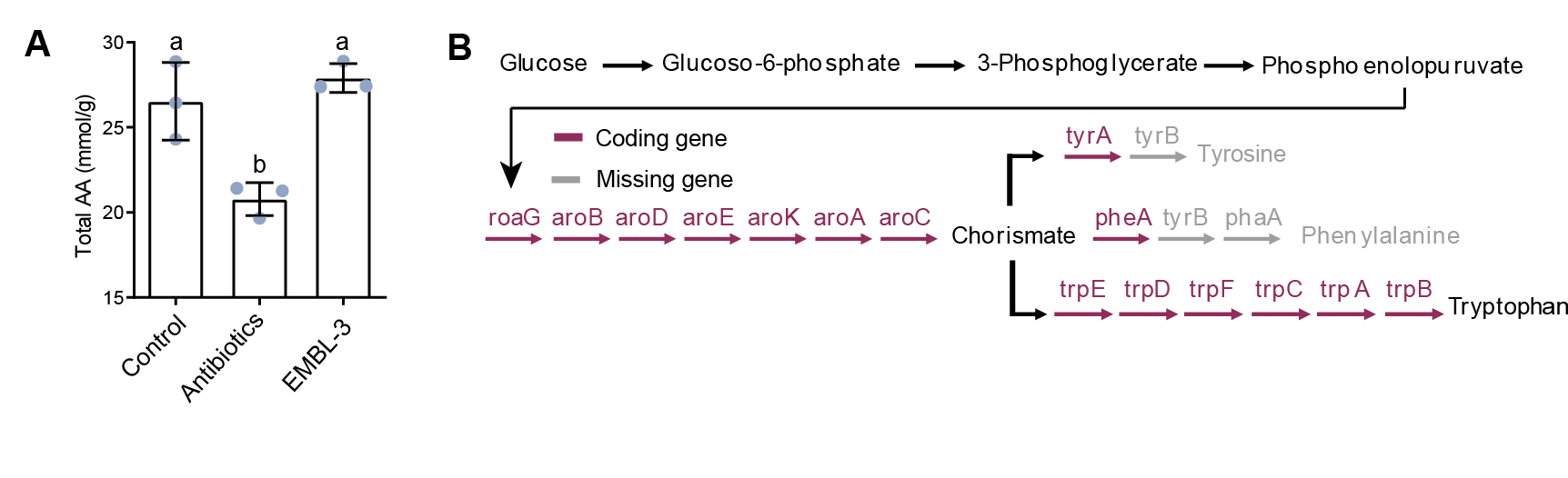


**Figure S3 The total amino acid content in FAWs (A) and the aromatic amino acid synthesis gene cluster of EMBL-3 (B).**

Note: Data are mean ± SEM, The statistical analysis was based on multiple comparisons. Different lowercase letters indicate significant differences among the treatments (*P* < 0.05).


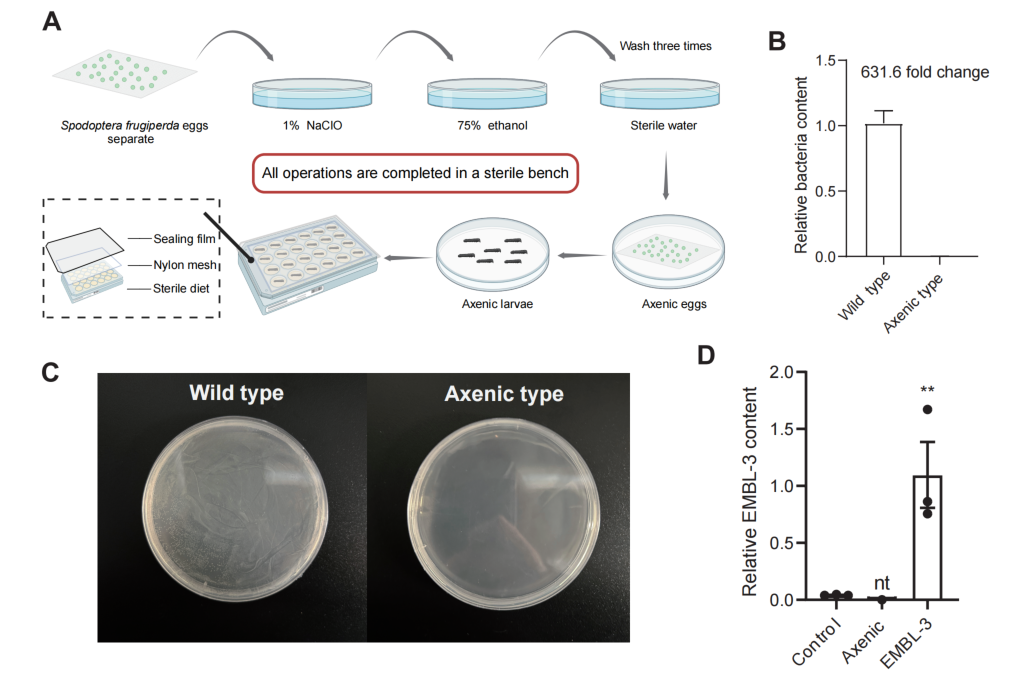


**Figure S4 Workflow for generating axenic FAWs.**

Note: (a) Workflow. (b) Relative bacterial abundance in axenic and wild-type FAWs. (c) Symbiont colonies from axenic and wild-type FAWs. (d) EMBL-3 content under different treatment. Data are mean ± SEM, where “**” indicate significant differences *P* < 0.01, nt: not detected.


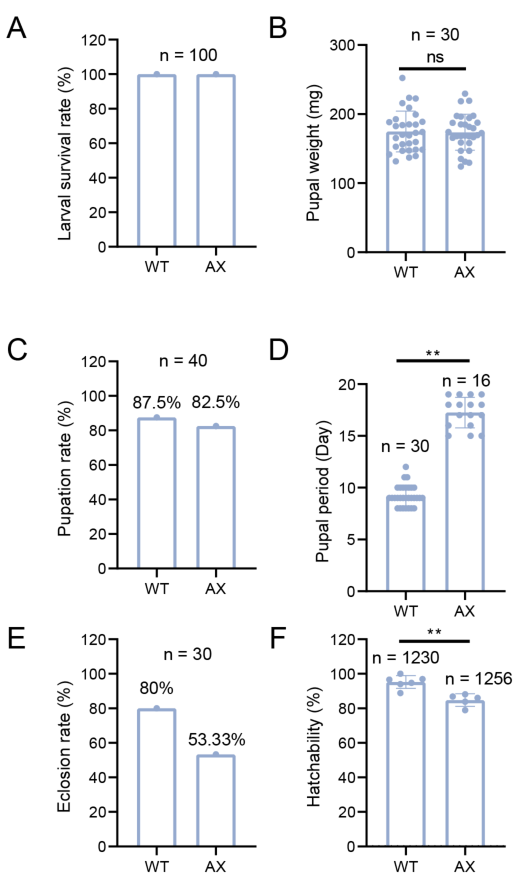


**Figure S5 Differences in growth, development and reproduction between wild type FAWs (WT) and sterile FAWs (AX).**

**Note:** Data are mean ± SEM The statistical analysis was based on *t*-test. Where “*” and “**” indicate significant differences *P* < 0.05 and *P* < 0.01, respectively, ns, not significant


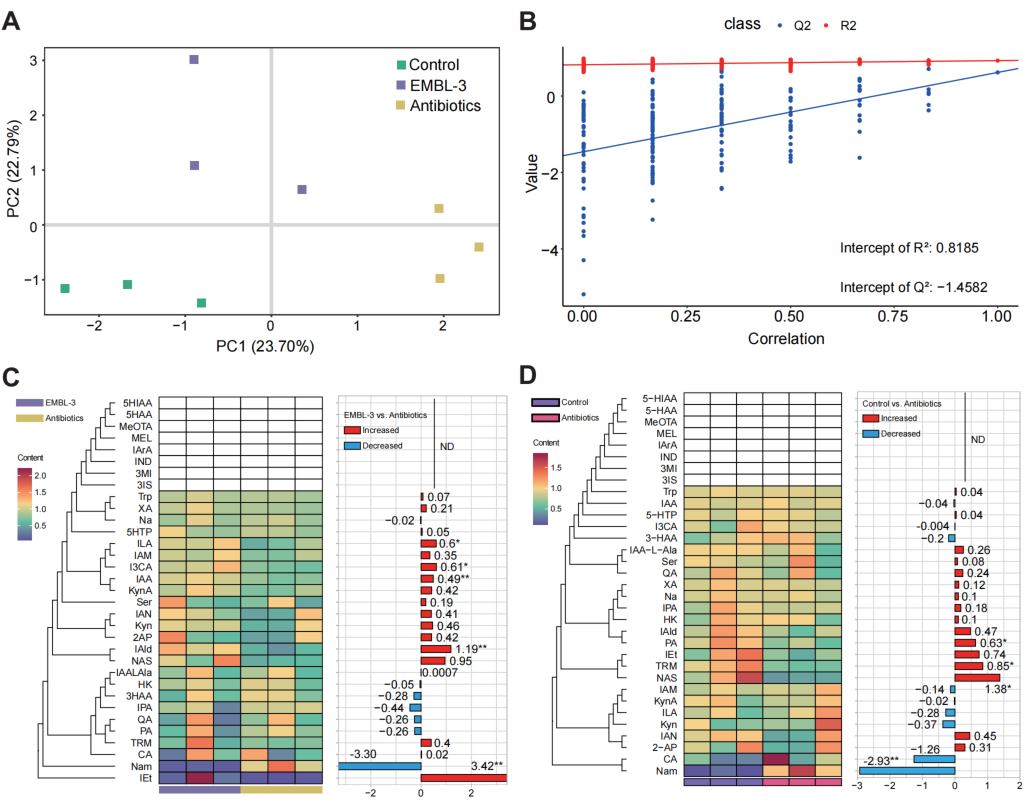


**Figure S6 Differences and consistency of tryptophan targeted metabolome in different treatments of FAWs.**

Note: A: Principal component analysis of the tryptophan targeted metabolomic profiles of control, antibiotics treatment, and EMBL-3 fed group. B: OPLS-DA analysis of c profiles of control, antibiotics treatment, and EMBL-3 fed group. C and D: metabolites difference between control and antibiotics group or EMBL-3 and antibiotics group. The left heatmap represents the metabolite patterns normalized by rows, while the right figure shows the results of the significance analysis based on the t-test. The bar graphs represent the *P* values, and the * indicate significant differences (*P* < 0.05).


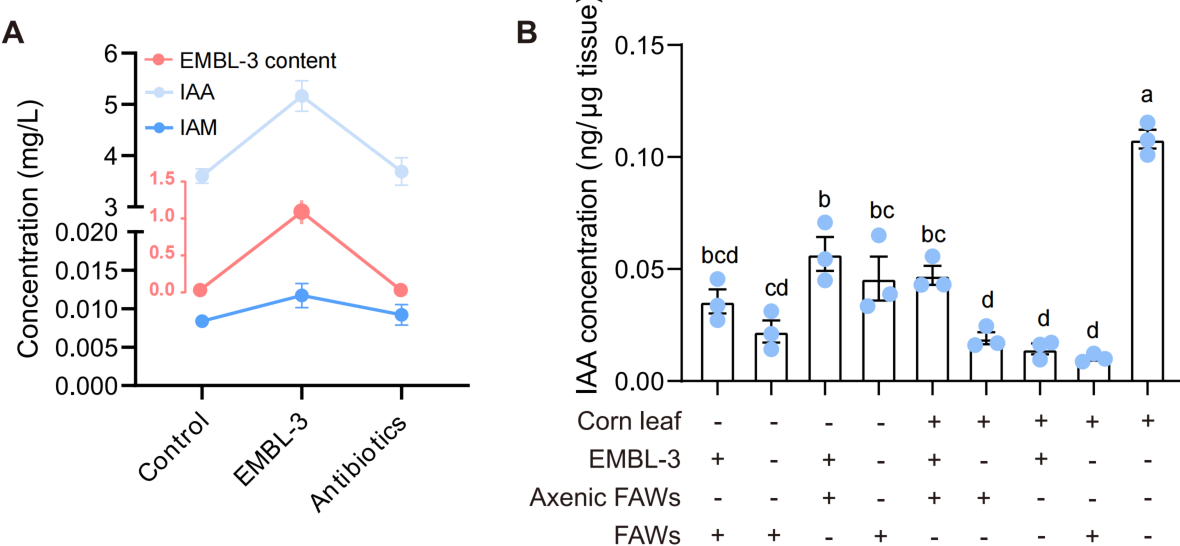


**Figure S7 The influence of EMBL-3 abundance on the content of IAA and IAM.**

Note: (a): IAA, IAM, and EMBL-3 content in control, EMBL-3, and antibiotics group, IAA and IAM concentration based on the tryptophan targeted metabolome. (b): IAA concentrations of different treatment. Data are mean ± SEM. The statistical analysis was based on multiple comparisons. Different lowercase letters indicate significant differences among the treatments (*P* < 0.05).


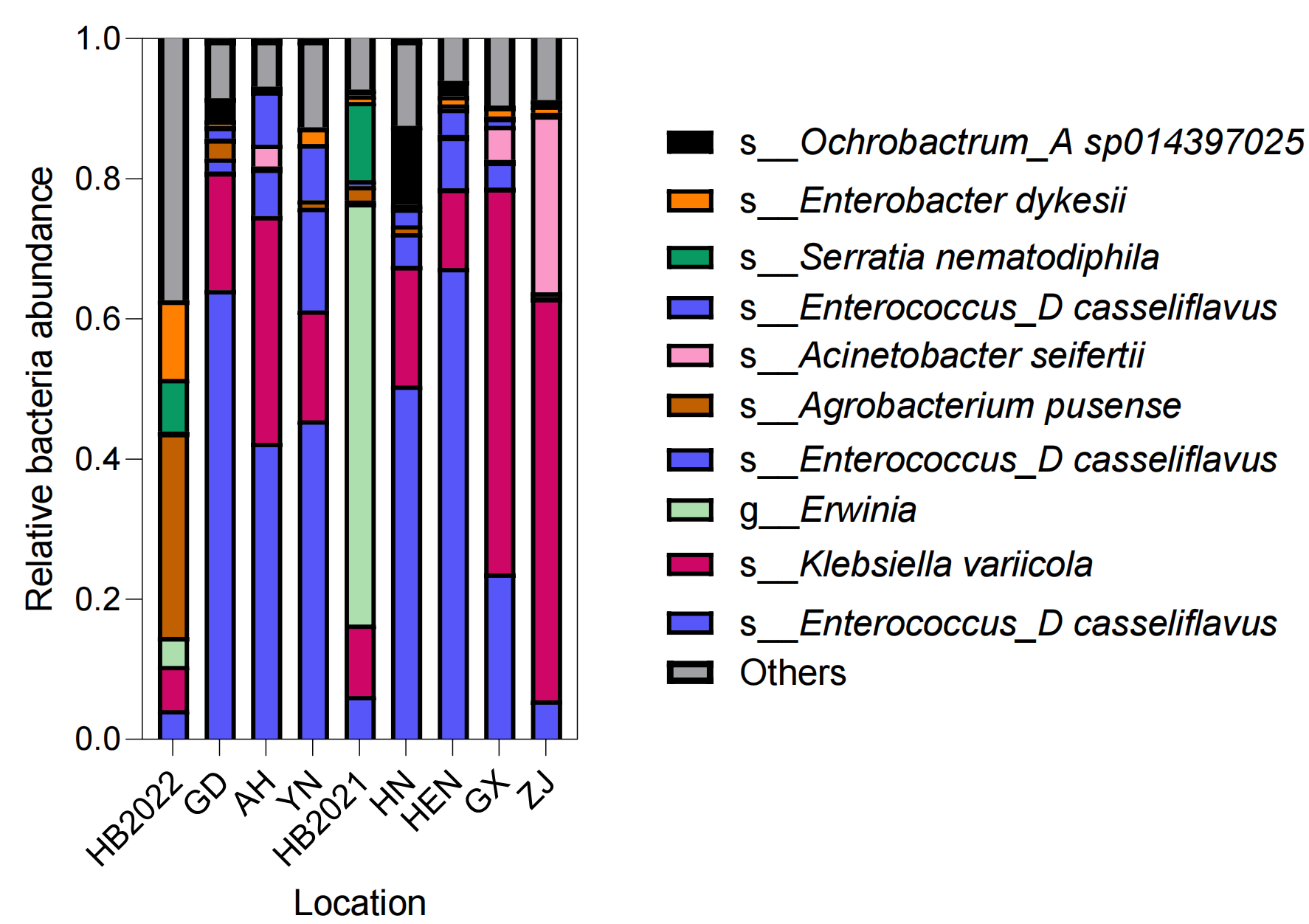


**Figure S8 Relative abundance of top 10 MAGs among 9 field population of FAWs.**


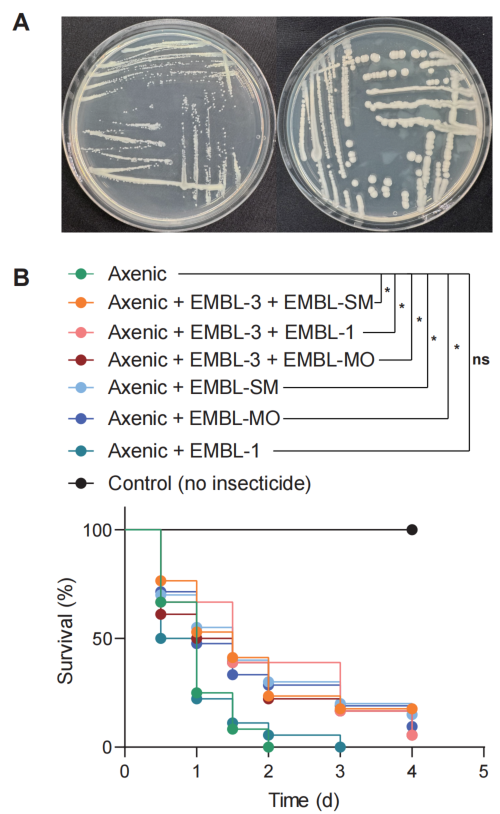


**Figure S9 The influence of EMBL-1 and EMBL-MO on the insecticide susceptibility of FAWs.**

Note: A: Symbiont colonies of EMBL-1 (left) and EMBL-MO (right). B: Effect of EMBL-1 and EMBL-MO on insecticide susceptibility in wild-type and axenic FAWs. Survival curve significance was determined by the log-rank (Mantel–Cox) test, where “*” indicate significant differences *P* < 0.05, ns, not significant.


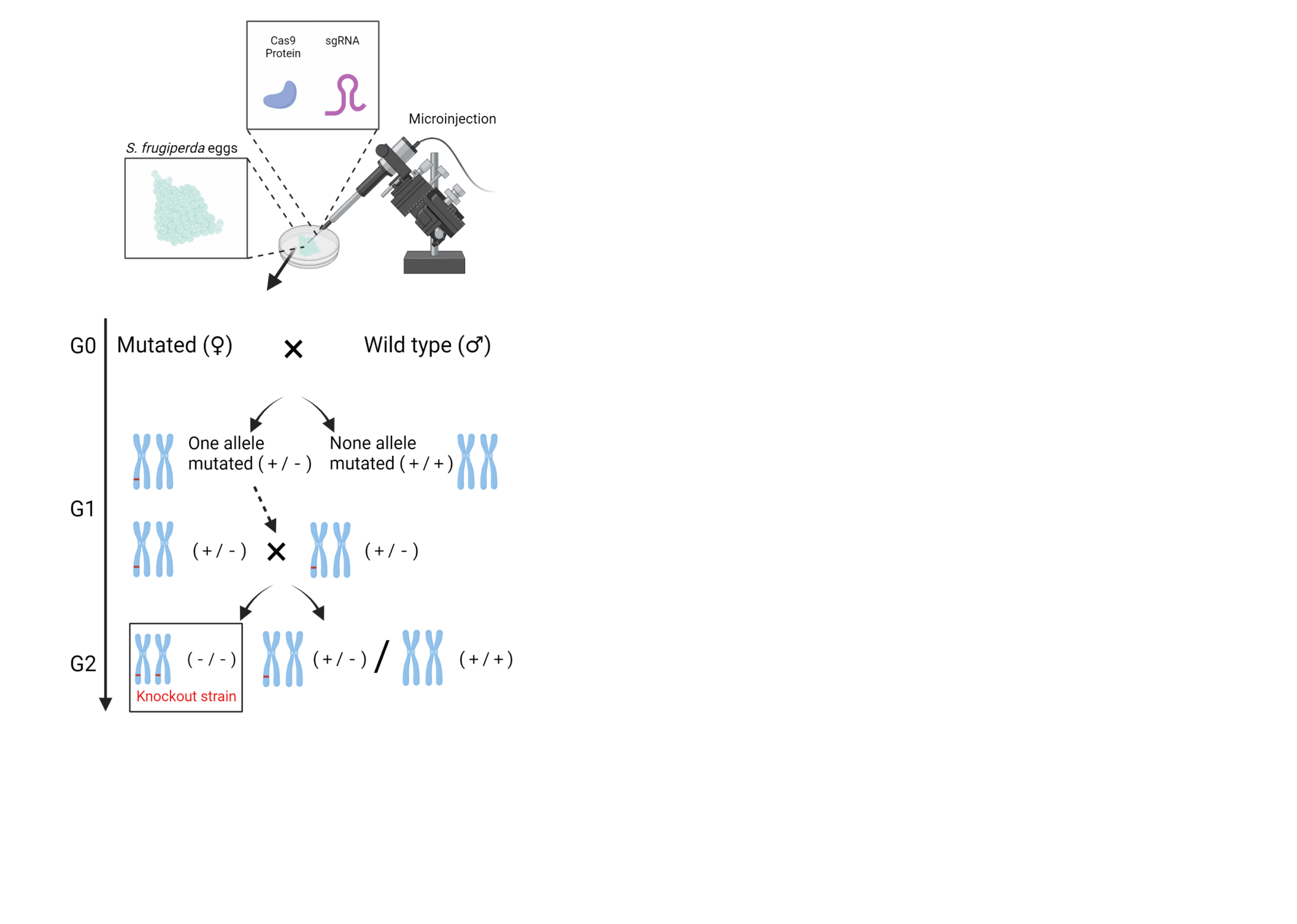

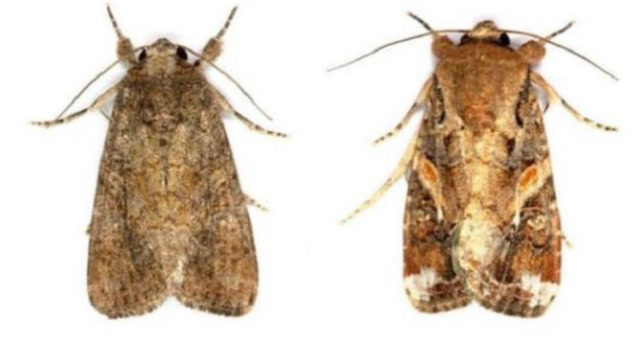

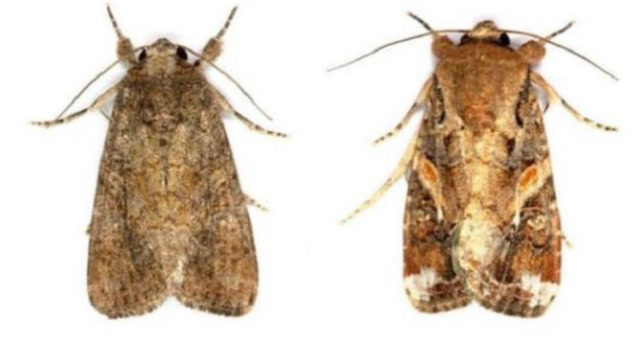


**Figure S10 Workflow for CRISPR/Cas9-mediated *UGT2* knockout in FAWs.**


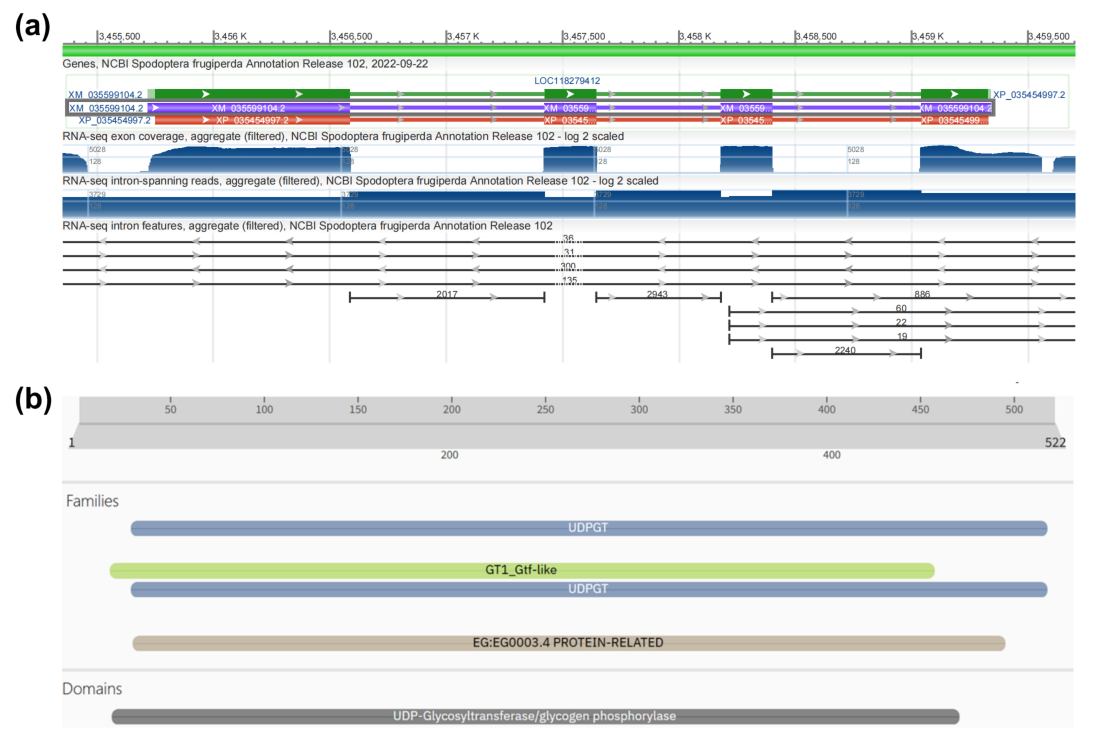


**Figure S11 Gene structure (up) and** **coding protein domain predicted (down) of *UGT2*.**

Note: the gene exons and transcript were download form NCBI database, the protein domain was predicted through InterProw (https://www.ebi.ac.uk/interpro).


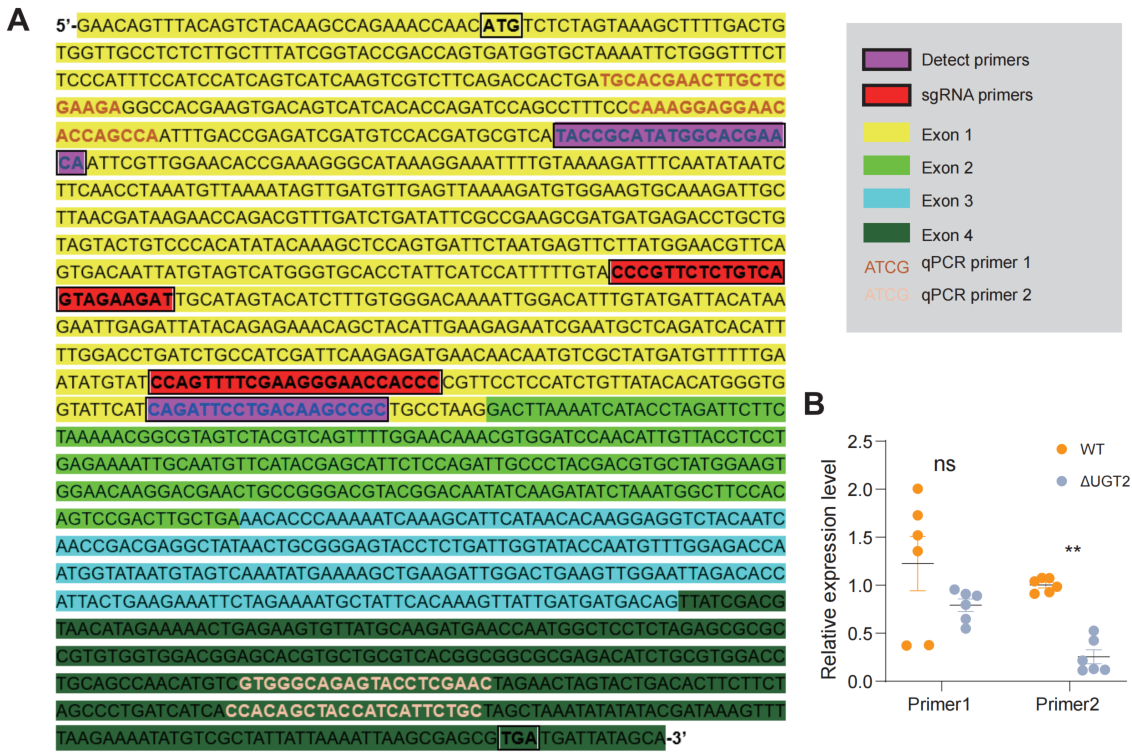


**Figure S12 Gene sequence, exon, and primer position information of *UGT2* (A). The mRNA expression level of *UGT2* in wild-type and *UGT2*-knockout strains FAWs (B).** Significance difference was determined by the *t*-test, where “**” indicate significant differences *P* < 0.01, ns, not significant.
